# Phase I trial of intracerebral convection-enhanced delivery of carboplatin for treatment of recurrent high-grade gliomas

**DOI:** 10.1371/journal.pone.0244383

**Published:** 2020-12-29

**Authors:** Joshua L. Wang, Rolf F. Barth, Robert Cavaliere, Vinay K. Puduvalli, Pierre Giglio, Russell R. Lonser, J. Bradley Elder

**Affiliations:** 1 Department of Neurological Surgery, The Ohio State University College of Medicine Wexner Medical Center, Columbus, Ohio, United States of America; 2 Department of Pathology, The Ohio State University College of Medicine Wexner Medical Center, Columbus, Ohio, United States of America; 3 Division of Neuro-Oncology, Department of Neurology, The Ohio State University College of Medicine Wexner Medical Center, Columbus, Ohio, United States of America; National Cancer Institute, UNITED STATES

## Abstract

**Background:**

Carboplatin is a potent cytoreductive agent for a variety of solid tumors. However, when delivered systemically, clinical efficacy for the treatment of high grade gliomas is poor due to limited penetration across the blood-brain barrier (BBB). Direct intracerebral (IC) convection-enhanced delivery (CED) of carboplatin has been used to bypass the BBB and successfully treat the F98 rat glioma. Based on these studies, we initiated a Phase I clinical trial.

**Objective:**

This Phase I clinical trial was conducted to establish the maximum tolerated dose and define the toxicity profile of carboplatin delivered intracerebrally via convection enhanced delivery (CED) for patients with high grade glial neoplasms.

**Methods:**

Cohorts of 3 patients with recurrent WHO grade III or IV gliomas were treated with escalating doses of CED carboplatin (1–4 μg in 54mL over 72 hours) delivered via catheters placed at the time of recurrent tumor resection. The primary outcome measure was determination of the maximum tolerated dose (MTD). Secondary outcome measures included overall survival (OS), progression-free survival (PFS), and radiographic correlation.

**Results:**

A total of 10 patients have completed treatment with infusion doses of carboplatin of 1μg, 2μg, and 4μg. The total planned volume of infusion was 54mL for each patient. All patients had previously received surgery and chemoradiation. Histology at treatment include GBM (n = 9) and anaplastic oligodendroglioma (n = 1). Median KPS was 90 (range, 70 to 100) at time of treatment. Median PFS and OS were 2.1 and 9.6 months after completion of CED, respectively. A single adverse event possibly related to treatment was noted (generalized seizure).

**Conclusions:**

IC CED of carboplatin as a potential therapy for recurrent malignant glioma is feasible and safe at doses up to 4μg in 54mL over 72 hours. Further studies are needed to determine the maximum tolerated dose and potential efficacy.

## Introduction

Current treatment for newly-diagnosed glioblastoma (WHO grade IV astrocytoma, GBM) includes surgical resection followed by concurrent radiation and temozolomide with subsequent adjuvant temozolomide [1]. Despite maximal therapy, these tumors invariably recur and the disease is nearly uniformly fatal with median overall survival of 15 months. Further surgery and chemotherapy provide only modest benefits in survival and quality of life. Despite the development of potent therapeutic agents *in vitro*, the clinical effectiveness of these agents for the treatment of high grade gliomas has not been realized due to poor penetration across the blood-brain barrier (BBB), significant toxicity with systemic administration [2] or reliance on diffusion based distribution [3]. Direct IC administration by means of convection-enhanced delivery (CED) via catheters placed into the tumor and/or surrounding brain can be used to bypass the BBB and deliver low and high molecular weight therapeutic agents [4–7] over large targeted regions of the central nervous system (CNS).

Carboplatin is a highly effective anti-cancer drug that has been used to treat a wide variety of malignancies [8]. Pre-clinical *in vitro* and animal studies have shown that gliomas are sensitive to this agent, but systemic administration of platinum drugs to treat brain tumors in humans has been limited by systemic toxicity and inability to penetrate an intact BBB in effective concentrations [2]. Animal studies have demonstrated significant therapeutic efficacy using the F98 rodent glioma model with CED of carboplatin either alone or in combination with radiation therapy [9–11]. This tumor model resembles human high grade gliomas in a number of important ways, including its invasive pattern of growth, weak immunogenicity, and lethality with as few as 100 tumor cells. Dose-escalation studies of CED of carboplatin in non-tumor bearing rats and in primate brainstems have shown minimal toxicities at lower dose ranges [12, 13]. To assess the feasibility and safety of CED of carboplatin for treatment of recurrent high grade gliomas, a Phase I trial was performed of recurrent malignant glioma patients that underwent infusion of this platinated anti-glioma agent. Interim analysis of the data through the first two cohorts was analyzed upon completion of all study timepoints and is presented here.

## Methods

### Patient eligibility criteria

**Fig 1:**
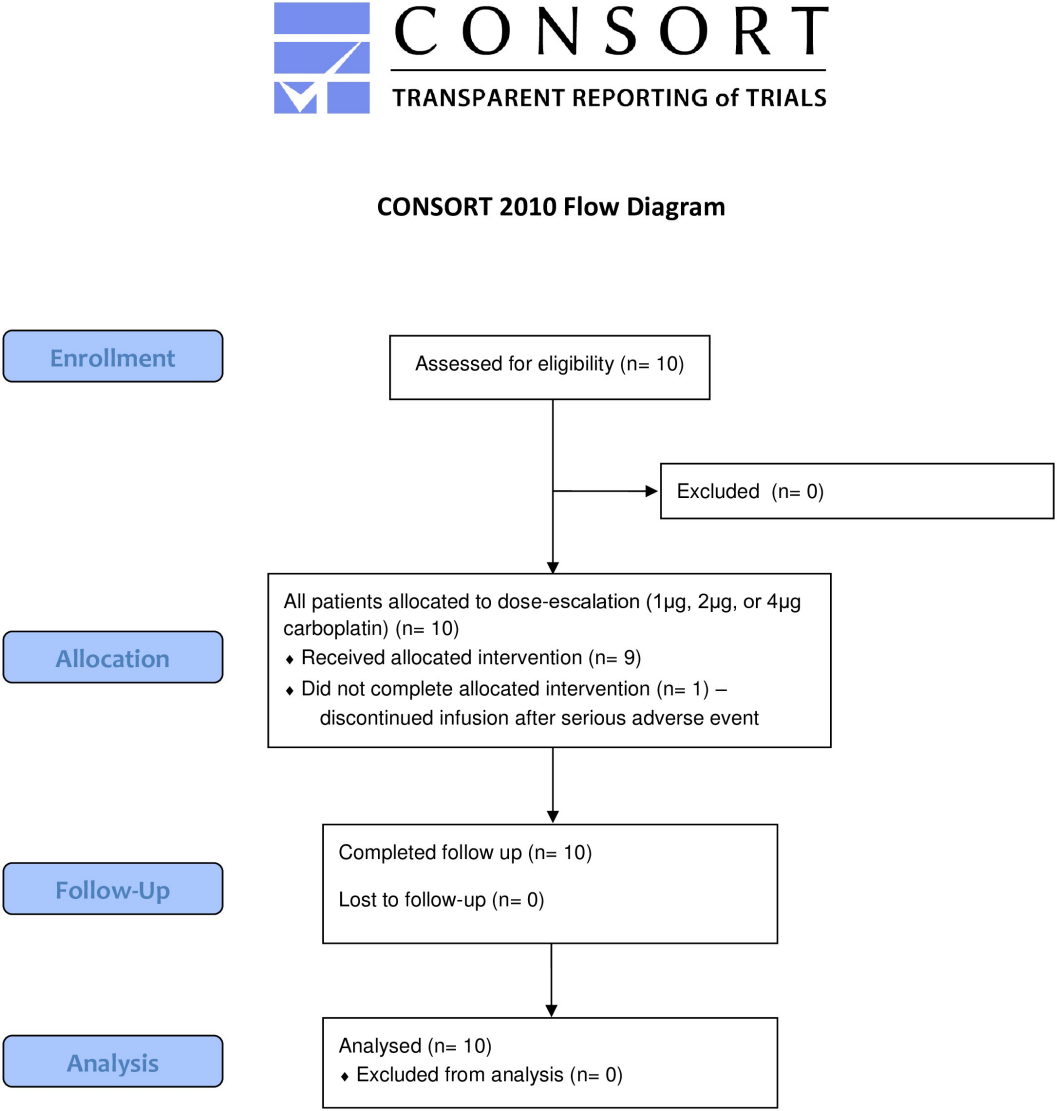
CONSORT flow diagram.

Patients were recruited at The Ohio State University James Cancer Hospital between July 2012 and December 2017. Clinic patients were referred to Tumor Board after presentation to Neuro-Oncology clinic with radiographic progression of disease, and subjects were considered for the trial during these weekly Tumor Board discussions with faculty of the neuro-oncology, neurosurgery, and radiation oncology teams. Patients with histologically confirmed supratentorial WHO grade III astrocytoma, oligodendroglioma, or grade IV glioblastoma with progressive disease, for which craniotomy and repeat resection was recommended were considered for this trial. All patients previously underwent radiation therapy and cytoreductive chemotherapy, and patients who previously received bevacizumab were eligible. Patients with multifocal disease, cerebral spinal fluid disseminated tumor, or tumor within 2 cm of the ventricles were excluded from the trial. Full inclusion and exclusion criteria, as well as the complete trial protocol, are listed in S1 Appendix. This study was carried out with the approval of the Institutional Review Board at The Ohio State University (ClinicalTrials.gov identifier: NCT01644955; OSU protocol: OSU-10151). The clinical trial (and all other appropriate therapies) was fully explained to all patients including all risks of the clinical trial, and informed consent was obtained from all patients. The study was carried out and reported in accordance with the TREND statement (Fig 1 and S2 Appendix).

### Follow-up

Patients were assessed in the outpatient setting every 4 weeks for the first 16 weeks and every 8 weeks thereafter until 30 weeks following surgery and carboplatin infusion. Evaluation included full general and neurologic examinations, mini-mental status examination (MMSE), and KPS scoring. Magnetic resonance (MR)-imaging was performed every 8 weeks and was evaluated by McDonald criteria. Toxicity was assessed according to the National Cancer Institute Common Terminology Criteria for Adverse Events, Version 4.0.

### Study design

This Phase I trial was designed as a traditional 3+3 open-label, nonrandomized dose-escalation study, in which groups of 3 patients were treated with escalating doses of carboplatin using CED. If one instance of dose-limiting toxicity (DLT) was observed among the initial 3 patients treated at a dose level, an additional 3 patients had to be treated at that dose level with no further DLT for dose escalation to proceed. The maximum tolerated dose (MTD) was the highest dose to cause DLT in no more than 1 of 6 patients at that dose level. Because a traditional 3+3 dose-escalation design was chosen, no statistical power analyses were required or performed [14].

**Fig 2:**
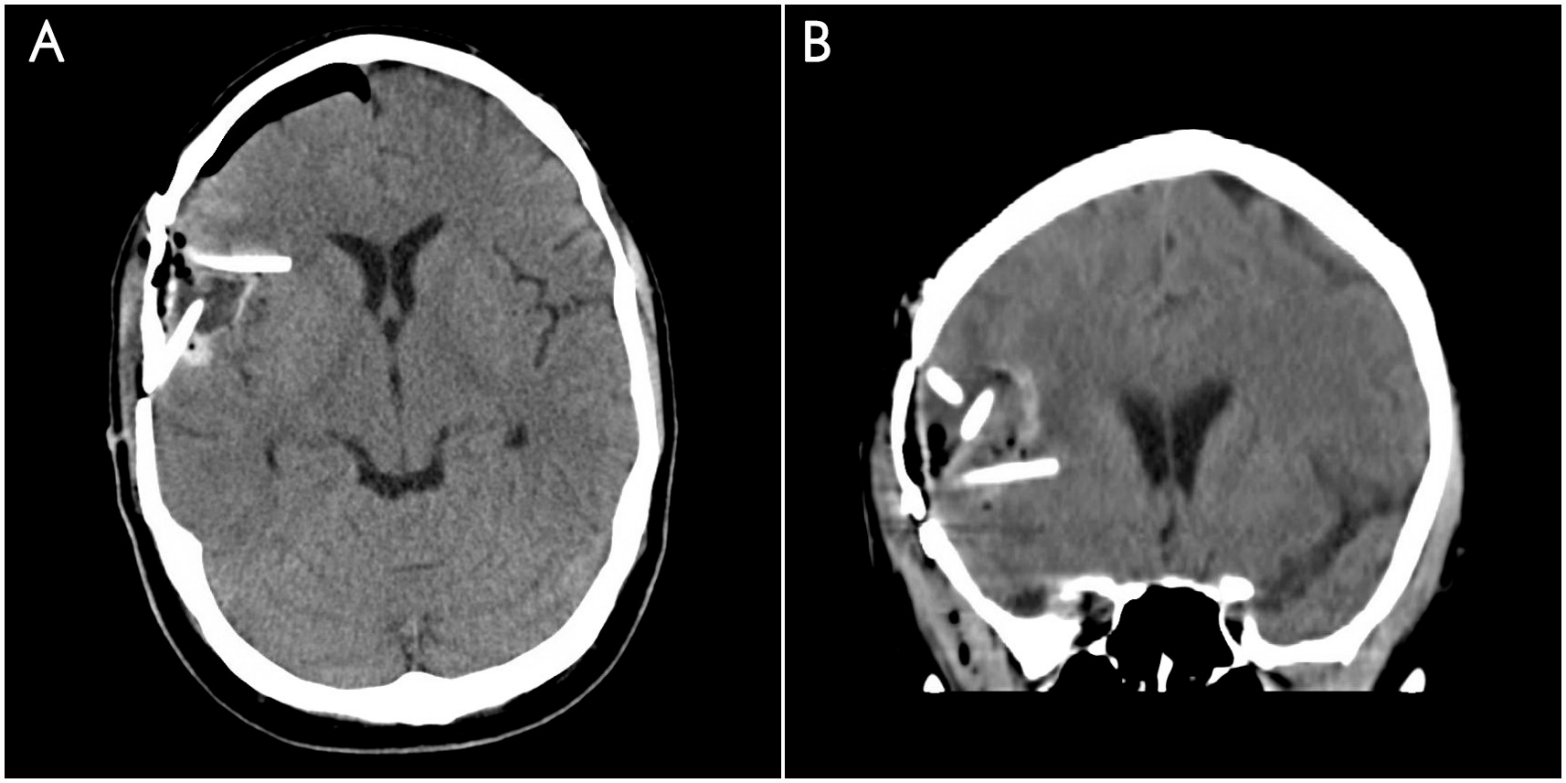
Postoperative imaging. Representative (A) axial and (B) coronal planes of a CT head on postoperative day 0 demonstrating postsurgical changes and three catheters traversing the lesion and entering the surrounding parenchyma.

Patients enrolled in the study underwent repeat craniotomy for tumor resection. Following intraoperative confirmation of recurrence by histopathologic frozen section, 1 to 4 catheters (Medtronic Becker EDMS Ventricular Catheter 20cm Barium Impregnated cerebrospinal fluid ventricular catheters; Medtronic, Inc., Minneapolis, Minnesota) were inserted stereotactically into the area surrounding the resection cavity (Fig 2). Carboplatin infusion commenced following postoperative computed tomography (CT) confirmation of catheter placement. Carboplatin diluted in 0.9% saline in a total volume of 54 mL (isotonic final solution) was administered IC by CED at a flow rate of 0.75 mL/hour over 72 hours (concentration, 0.019 μg/mL and 0.037 μg/mL) using a syringe pump. The total flow rate was divided among the catheters placed. The catheters and syringe pumps were attached to a bedside programmable syringe-type microinfusion pump (Medex/Medfusion 3500 Syringe pump, Smiths Medical, Minneapolis, Minnesota) via infusion tubing (Vygon Neuro Infusor Infusion catheter system, Vygon USA, Lansdale, Pennsylvania). The catheters were removed after completion of infusion and patients underwent post-infusion MR-imaging. The Food and Drug Administration (FDA) mandated starting dose of carboplatin was 1 μg over 72 hours for the first 3 patients enrolled. The dose was escalated to 2 μg over 72 hours for patients #4–7 and to 4 μg for patients #8–10.

All adverse events during the patient’s clinical course were recorded, and the investigators discussed to what degree the study interventions could have caused each adverse event. DLTs were defined, in the presence of supportive care, as any Grade 3 or 4 toxicity that is possibly, probably, or definitely related to the study intervention within 28 days of the intervention. Non-hematologic toxicities such as rash, nausea, vomiting, and hypertension were only considered DLTs if they remained grade 3 or greater despite maximal medical therapy 72 hours after maximal medical therapy was initiated.

Interim analysis was performed after all patients enrolled in dose levels 1 through 3 had completed all follow-up, as per recommendation from the IRB.

### Statistical analysis

Primary outcome measure was MTD. Secondary outcome measures were overall and progression free survival. Demographic and baseline characteristics were recorded as medians for continuous variables and as proportions for categorical variables. All adverse events occurring within the first 28 days after carboplatin infusion and survival metrics were analyzed by intended dose group. Statistical analyses were performed using JMP 11 (SAS Institute Inc., Cary, NC, USA).

## Results

### Patient characteristics

A total of 10 patients (7 male, 3 female) were enrolled and received treatment in this single-center phase I clinical trial between July 2012 and October 2017. Nine patients were enrolled at time of first recurrence and 1 at second recurrence. The demographics and clinical characteristics of the patients are listed in Table 1. No patients were lost to follow up, excepting those who expired. Tumor and catheter placement locations are detailed in Table 2.

**Table 1:** Patient demographics and clinical characteristics at trial enrollment.

Patient	Sex	Age, years	KPS, %	Histopathology	Time from first diagnosis to study treatment, months	Prior surgeries	Prior radiotherapy	Prior systemic therapies
1	F	33	90	GBM	115	1	1	1
2	M	33	100	GBM	9	2	1	1
3	M	49	80	GBM	10	1	1	2
4	M	29	90	Grade III oligodendroglioma	18	1	1	2
5	F	30	100	GBM	21	1 (biopsy)	1	1
6	M	62	80	GBM	69	1	1	3
7	M	60	70	GBM	7	1	1	1
8	F	55	100	GBM	24	1	1	1
9	M	62	80	GBM	27	1	1	1
10	M	55	90	GBM	9	1	1	1

**Table 2:** Tumor location and catheter placement.

Patient	Tumor location	# of catheters	Catheter location with respect to resection cavity
1	Left frontal	2	anterior; anterior superior
2	Right frontal	3	anterior; anterior superior; deep
3	Right frontal	2	anterior; posterior
4	Left frontal	2	anterior inferior; lateral
5	Left occipital	2	posterior; posterior superior
6	Right frontal	2	posterior inferior; superior
7	Right frontoparietal	2	posterior; superior
8	Right parietooccipital	2	posterior medial; anterior inferior
9	Right frontal	2	posterior; superior
10	Left frontal	2	anterior superior; posterior superior

### Anti-tumor response

All patients were assessed for anti-tumor response. At the time of analysis, 9 of 10 patients were deceased. Survival statistics are summarized in Table 3. Median PFS was 2.1 months, and PFS at 6 months post-infusion was 20%. Median OS was 9.6 months, and 12-month OS was 40%.

**Table 3:** Patient survival outcomes.

	Total carboplatin dose (μg)		Age (years)		Survival after treatment (months)	
Patient	Planned	Received	At diagnosis	At treatment	Progression-free	Overall
1	1	1	24	33	1.9	9.6
2		1	32	33	0.8	6.3
3		1	48	49	1.0	1.7
4	2	2	29	30	10.8	17.3
5		2	56	62	1.9	10.8
6		1.3	28	29	2.2	27.5
7		2	60	60	1.6	4.9
8	4	4	53	55	3.7	23.9
9		4	60	62	2.5	3.4
10		4	54	55	9.2	

### Imaging findings

In all patients, there were no new areas of restricted diffusion to suggest ischemic event related to infusion. No new cystic areas or other complications potentially associated with surgery or CED were found on follow up imaging.

### Dose escalation and dose-limiting toxicity

None of the reported adverse events were considered probable or definitely related to the study treatment. A total of 9 adverse events were reported, of which 4 were Grade 3 or higher (summarized in Table 4). A single adverse event possibly related to study treatment was reported. Patient 6 experienced an isolated grade 2 generalized tonic-clonic seizure complicated by grade 4 hypoxia resulting from lorazepam administration on the second night of infusion. Carboplatin infusion was halted, and the IC catheters were removed (he received a total dose of 1.3 μg of carboplatin). The patient recovered to baseline neurologic and medical status within 24 hours. No dose-limiting toxicities were observed in any of the other patients. Because the infusion was halted in this patient, a fourth patient was enrolled at dose level 2. Patient 10 experienced two self-limited simple partial seizures that did not generalize on post-operative days 0 and 1, which were treated with lorazepam, levetiracetam, and lacosamide. He recovered to baseline neurologic and medical status, and thus infusion was continued and he completed the planned infusion of 4 μg.

**Table 4:** Reported adverse events (grade ≥ 2).

Patient	Dose planned	Dose received	POD #	Event	Grade	Relation to treatment
3	1 μg	1 μg	28	Gait disturbance	2	Unrelated
				Muscle weakness (Left sided)	2	Unrelated
5	2 μg	2 μg	157	Encephalopathy	3	Unrelated
6	2 μg	1.3 μg	2	Ventricular tachycardia	2	Unlikely
				Seizure	2	Possible
				Hypoxia	4	Unlikely
7	2 μg	2 μg	14	Thromboembolic event	2	Unlikely
			16	Cerebral edema	4	Unlikely
			51	Wound dehiscence	4	Unlikely
9	4 μg	4 μg	0	Right eye swelling	2	Unrelated
			1	Dysphagia	3	Unrelated
			55	Muscle weakness (Left sided)	2	Unrelated
			76	Urinary tract infection	3	Unrelated
10	4 μg	4 μg	0	Seizure	2	Possible
			13	Nausea	2	Unrelated
			27	Neutropenia	3	Unrelated

POD: post-operative day.

Within 4 weeks following surgery and infusion, 2 additional patients experienced partial seizures. Both these patients had occasional seizures before enrollment and the exact relationship between these seizures and the study treatment is unclear. Patient 1 reported improvement in seizure frequency and severity following treatment compared to pre-enrollment. Patient 2 reported higher frequency of seizures in the month following treatment and these were controlled with an increase in steroid dose. There were no treatment-related deaths.

## Discussion

Since the early 1990s, platinated chemotherapies have not been used routinely in the treatment of malignant gliomas, following the results of a European Organization for Research and Treatment of Cancer randomized controlled trial of 285 malignant glioma patients that failed to demonstrate difference in PFS or OS between those treated with cisplatin and radiotherapy in combination or with radiotherapy alone [15]. Despite producing significant systemic toxicity, both the oral and intravenous routes of administration of platinated chemotherapies have not resulted in therapeutic concentrations of platinated drugs in the CNS [2].

Several approaches have been proposed to bypass the BBB and deliver anti-cancer drugs directly to the brain, thereby increasing tumor drug concentrations and reducing the associated systemic toxicity. These methods include intratumoral bolus injection [16], surgical implantation of drug-loaded polymer matrices [16–20], and CED of drugs via catheters placed into the tumor and/or surrounding brain [4, 21]. Compared to diffusion of a drug from polymer matrices, convection (or “bulk” flow) is independent of the drug’s molecular weight, ionic charge, or concentration gradient [7]. Both low and high molecular weight agents can be delivered via CED effectively and homogeneously to large volumes of the brain without significant functional or structural damage [22, 23].

Using the F98 rodent model, Elleaume, Barth, and colleagues determined that the intra-tumor carboplatin concentration following CED of 20 μg was equivalent to that observed following intravenous administration of a lethal dose of 25 mg which was 1,000 fold greater than the amount of drug administered by CED [10]. Furthermore, rats bearing small tumors had longer survival times and cure rates compared to those bearing large tumors following treatment with either carboplatin alone or in combination carboplatin and radiation therapy [10]. This underscores the importance of tumor resection to reduce overall tumor burden prior to administering carboplatin via CED.

Recent clinical reports suggested that direct IC administration of platinated drugs was well tolerated [19, 21, 24]. In a case report, Barua and colleagues infused 27.9 mL of carboplatin per day (at an initial concentration of 0.18 mg/ml, and up to 0.24mg/ml after four weeks of implantation, total 5.02–6.70 mg of carboplatin per day) over multiple days using a transcutaneous port for CED and observed a significant reduction in tumor volume as determined radiologically [24]. They did not report any adverse effects with the treatment, and their patient survived 8 months following implantation of CED catheters and 33 months after initial diagnosis of their GBM.

To the best of our knowledge, this Phase I trial is the first clinical trial to demonstrate that CED of carboplatin is safe and feasible up to doses of 4 μg for adult patients with recurrent high-grade glioma. Immediate IC infusion of carboplatin following resection of recurrent or progressive tumor may provide increased local control, bridging the gap between surgery and resumption of systemic therapies. Furthermore, the results described here show that the placement of IC catheters and surgical resection in a single operation and direct IC infusion of chemotherapy, was safe and not associated with adverse events in this cohort of patients. Unfortunately, patient accrual was slower than anticipated, and as such the institutional IRB recommended pausing the trial for analyses, which have been presented here. Although all the proposed doses up to 128 μg were not able to be tested due to slow accrual and early pausing of the trial, there were no DLTs up through the 4 μg dose group.

In comparison to preclinical rodent studies, the carboplatin infusate concentration, total dose infused, and flow rates were lower after accounting for differences in brain size and volume. These limitations in starting dose were mandated by the FDA during the IND application phase, and were based on preclinical primate data involving carboplatin CED into primate brainstems [25]. Using the F98 glioma model, rats received 20μg of carboplatin in 10μL (equivalent to 2mg/mL) to one cerebral hemisphere (average weight 600mg), yielding 2μg of carboplatin per 60mg per cerebral hemisphere [10]. The human brain is approximately 1000 times larger than the rat brain (600g per cerebral hemisphere). The highest doses proposed and actually used in this trial were 128μg and 4μg in 54mL (2.37μg/mL and 37ng/mL), respectively. In animal studies, rats were infused 10μL of drug over 30 minutes (0.33 μL/g/h), whereas in this trial patients were infused 54 mL into a 600g hemisphere over 72 hours (1.25 μL/g/h). Preclinical trials investigating safety of CED of carboplatin reported toxicities at 100μg in rat brains (600g per hemisphere) and dose-dependent neurotoxicity above 250–400μg in cynomologus monkeys.

No systemic toxicities associated with carboplatin administration (including bone marrow suppression leading to anemia, leukopenia, or thrombocytopenia, or gastrointestinal upset) were observed in the patients in this study. However, Patient 10 did have neutropenia on post-operative day 27. This was deemed unrelated to treatment since the patient was taking a number of medications associated with neutropenia, including phenytoin. This lack of observed toxicities was likely due in part to the relatively low doses of carboplatin administered to these patients. Further dose escalation is warranted to determine the systemic and local toxicity profiles of carboplatin via CED. Since a constant infusion volume was used across all dosing levels, further dose increases of carboplatin could be achieved without increasing total infusion volumes or rates.

Seizures were the most common pre-trial complaint among enrolled subjects, but in both cases, the patients reported an improvement of seizure frequency and severity following the study treatment. Any improvements in seizure activity in the immediate postoperative period most likely were due to the rapid decrease in mass effect afforded by surgical resection, as well as surgical removal of epileptogenic tissue. Patient #6 was the only patient to experience an adverse event while receiving CED infusion of carboplatin. The generalized seizure he experienced was classified as possibly related to the trial treatment, but it remains unclear whether this event was due to the infusion or could have been anticipated based on his partially controlled seizure history.

Patient #7 required readmission for placement of an external ventricular drain (EVD) due to cerebral edema that was determined unlikely due to treatment given the time between CED and readmission and the fact that his steroid medications had been abruptly discontinued inadvertently at his rehabilitation facility. His neurologic examination on admission was notable for significant lethargy and an external ventricular drain was placed due to presumed high intracranial pressure. Of note, the intracranial pressures were normal and the ventriculostomy was removed after 24 hours. His neurologic examination returned to baseline within 12 hours of restarting steroids. The same patient also presented with partial wound dehiscence 7 weeks after surgery. This was felt to be most likely related to bevacizumab treatment, which had started 4 weeks after surgery, rather than the clinical trial.

Efficacy was not the primary objective of this Phase 1 trial, conducted on a limited number of previously treated patients. Median OS was 292 days (9.7 months), and one-year survival was 29%, which compares somewhat favorably to previous trials using systemically administered carboplatin, in which median OS was around six months [26–28]. In contrast to this trial, Prados and colleagues reported significant myelotoxicity (~31%) in recurrent high grade glioma patients treated with systemic carboplatin [28]. The previous EORTC systemic cisplatin trial for supratentorial malignant gliomas failed to demonstrate any benefit of systemic cisplatin. Patient #4, with pathologically diagnosed WHO grade III oligodendroglioma, had much longer PFS and OS times compared to the median, and further study is needed to understand the differences in treatment effects of CED carboplatin between pathologic tumor grades.

The generalizability and applicability of this Phase 1 trial to a wider population with recurrent GBM is limited due to small cohort size and low doses of carboplatin infused. The inclusion and exclusion criteria of the trial may have led to a predominance of younger patients in this cohort and high baseline KPS that may have contributed to the lack of treatment-related adverse events and the trend towards more favorable survival compared to historical controls. CED is subject to a number of variables, such as backflow along the catheters or marked heterogeneity of drug distribution, though the use of multiple catheters mitigates some of this heterogeneity [29]. This trial thus far demonstrates the safety of CED in these patients and this data contributes to evidence regarding safety of direct infusion of cytotoxic agents into the brain.

## Conclusion

CED of carboplatin of up to 4 μg in a total volume of 54 mL for patients with GBM is a safe and feasible adjunct to re-resection of recurrent tumor. This treatment circumvents the BBB and provides immediate local adjuvant chemotherapy following surgery. Further study to define more clearly the toxicity and therapeutic efficacy IC CED of carboplatin used in this setting, as well as with further dose-escalation, and in combination with radiation therapy is warranted. Data presented here may support other investigators conducting trials incorporating CED and/or carboplatin now and in the future.

## Supporting information

S1 Appendix Clinical trial protocol. Complete clinical trial protocol approved by the Ohio State University IRB.
(DOCX)

S2 Appendix TREND checklist. Completed TREND checklist for this study.
(DOCX)
