# Supplementary material for: Phase I trial of intracerebral convection-enhanced delivery of carboplatin for treatment of recurrent high-grade gliomas
Source: PLoS One. 2020 Dec 29;15(12):e0244383. doi: 10.1371/journal.pone.0244383 (PMC7771668; doi:10.1371/journal.pone.0244383)
Supplement: S1 Appendix — Complete clinical trial protocol approved by the Ohio State University IRB. (DOCX) [file pone.0244383.s001.docx]

A CLINICAL TRIAL OF THE OHIO STATE UNIVERSITY

COMPREHENSIVE CANCER CENTER

**Intracerebral Convection-Enhanced Delivery of Carboplatin for Treatment of Recurrent High-grade Gliomas**

**PI:**

J. Bradley Elder, MD

Assistant Professor, Department of Neurosurgery

The Ohio State University Medical Center

Suite M410, Starling Loving Hall

320 W 10^th^ Ave.

Columbus, OH 43210

(614) 366-8327

**Sub-Investigators:**

Robert Cavaliere, M.D., Division of Neuro-Oncology, OSUMC

Herbert B. Newton, M.D., Division of Neuro-Oncology, OSUMC

IND #: 109979

Table of Contents

[1 - Objectives 4](#_Toc326746519)

[1.1 - Primary Objective 4](#_Toc326746520)

[1.2 - Secondary Objectives 4](#_Toc326746521)

[2 - Background and Study Rationale 4](#_Toc326746522)

[2.1 - Introduction 4](#_Toc326746523)

[2.2 - Glioblastoma multiforme 5](#_Toc326746524)

[2.3 - Carboplatin 6](#_Toc326746525)

[2.4 - Convection Enhanced Delivery 7](#_Toc326746526)

[2.5 - Pre-clinical Studies of Intratumoral Administration of Carboplatin and Cisplatin 7](#_Toc326746527)

[2.6 - Human Studies of Intracerebral Administration of Platinum Agents 14](#_Toc326746528)

[3 - Pharmaceutical Information 14](#_Toc326746529)

[4 - Patient Eligibility Criteria 15](#_Toc326746530)

[4.1 - Population 15](#_Toc326746531)

[4.2 - Inclusion Criteria 16](#_Toc326746532)

[4.3 - Exclusion criteria 17](#_Toc326746533)

[5 - Treatment Plan 18](#_Toc326746534)

[5.1 - Study Design 18](#_Toc326746535)

[5.2 - Proposed trial 18](#_Toc326746536)

[5.3 - Population 18](#_Toc326746537)

[5.4 - Outline of Treatment Plan 19](#_Toc326746538)

[5.5 - Pre-operative safety 20](#_Toc326746539)

[5.6 - Surgery 20](#_Toc326746540)

[5.7 - Carboplatin infusion 21](#_Toc326746541)

[5.8 - Assessments 21](#_Toc326746542)

[5.9 - Postoperative care and carboplatin infusion 22](#_Toc326746543)

[5.10 - Inpatient care 23](#_Toc326746544)

[5.11 - Post-discharge care / Outpatient monitoring 23](#_Toc326746545)

[5.12 - Dosage escalation plan 24](#_Toc326746546)

[5.12.1 - Dose escalation schedule 24](#_Toc326746547)

[5.12.2 – Dose escalation Justification 25](#_Toc326746548)

[5.13 - Dose-Limiting Toxicity (DLT) 26](#_Toc326746549)

[5.14 - Criteria for discontinuation of treatment 27](#_Toc326746550)

[6 - Regulatory and Reporting Requirements 27](#_Toc326746551)

[6.1 - Adverse Events (AE) 27](#_Toc326746552)

[6.1.1 - Adverse Event Definition 27](#_Toc326746553)

[6.1.2 - Attribution of an AE 27](#_Toc326746554)

[6.2 - Serious Adverse Event Reporting (SAE) 27](#_Toc326746555)

[6.2.1 - Non-serious adverse event definition 28](#_Toc326746556)

[6.3 - Unexpected Adverse Event 28](#_Toc326746557)

[6.4 - AE Reporting 28](#_Toc326746558)

[6.4.1 - Follow-up of AEs 28](#_Toc326746559)

[6.5 - Expected AEs that do not require expedited reporting 28](#_Toc326746560)

[6.6 - Investigator Reporting to the FDA 28](#_Toc326746561)

[6.7 - Data Saftey Monitoring Plan 29](#_Toc326746562)

[7 – Study Calendar 30](#_Toc326746563)

[8 - Clinical Response and Endpoint Definitions 31](#_Toc326746564)

[8.1 - Primary endpoint 31](#_Toc326746565)

[8.2 - Secondary endpoints 31](#_Toc326746566)

[8.2.1 - Neurological Examination Status 31](#_Toc326746567)

[8.2.2 - Radiographic Assessment compared to baseline MRI for patients with measurable disease 31](#_Toc326746568)

[8.2.3 - Evaluation of Best Overall Response 32](#_Toc326746569)

[8.2.4 - Confirmation of response 33](#_Toc326746570)

[9 - Statistical Considerations 33](#_Toc326746571)

[10 - References 33](#_Toc326746572)

[11 - Appendices 36](#_Toc326746573)

[Appendix A- Mini-Mental State Examination (MMSE) 36](#_Toc326746574)

# 1 - Objectives

The purpose of this phase I clinical trial is to begin translating the promising animal data into the clinical arena. The first step in this process is to establish the safety of this treatment strategy in humans.

1.1 - Primary Objective:

Establish the maximum tolerated dose and define the toxicity profile of carboplatin delivered intracerebrally via convection enhanced delivery (CED) for patients with high grade glial neoplasms. The maximum tolerated dose (MTD) of infused carboplatin may then be incorporated into future clinical studies.

1.2 - Secondary Objectives:

Examine the efficacy as defined by six-month progression free survival (PFS), median progression free sur­vival, over­all survival, and the radiographic response rate. Evaluate the drug distribution. These results will provide support for future clinical trials aimed at determining the efficacy of intracerebral carboplatin infusion either alone or in combi­nation with external beam X-irradiation.

# 2 - Background and Study Rationale

## 2.1 - Introduction

Glioblastoma multiforme (grade IV astrocytoma; GBM) is the most common primary central nervous system malignancy in adults and is nearly uniformly fatal[[1](#_ENREF_1)]. Current treatment strategies involve resection followed by radiation and chemotherapy. These tumors invariably recur despite maximal therapy, and further surgery and chemotherapy rarely yields more than modest benefit in terms of survival and quality of life. There are few options for patients with recurrent GBM. Despite significant research efforts over the last 20 years, the prognosis for patients diagnosed with GBM has improved only slightly, and average survival after diagnosis is only 12 – 15 months[[2](#_ENREF_2), [3](#_ENREF_3)].

Multiple properties of GBM contribute to the difficulty in developing effective adjuvant therapies. Chemotherapeutic agents are most commonly given by mouth or intravenous injection. However, the blood-brain barrier (BBB) limits the ability of chemotherapy agents to cross from the circulation into the brain to reach tumor cells, thus reducing the efficacy of systemic chemotherapy[[4](#_ENREF_4)]. Tumor invasiveness is another factor that contributes to overall malignancy[[5](#_ENREF_5)]. GBM tumor cells invade and migrate along white matter tracts, thus evading surgical removal and potentially avoiding the post-operative radiation field. Enlarging the radiation field could result in risks associated with radiation an increasing volume of normal brain parenchyma. New strategies are needed to treat GBM.

Convection enhanced delivery of chemotherapy may improve the delivery of chemotherapy to brain tumors by circumventing the BBB[[6](#_ENREF_6)]. This technique may also expose invasive cells not located near the primary tumor mass to the chemotherapeutic agent. Drugs that do not generally cross the BBB, but which are known to have anti-tumor efficacy may have a renewed role in brain tumor therapy using convection enhanced delivery.

With this proposal, we are requesting permission to administer carboplatin via convection enhanced delivery in patients with recurrent malignant glioma. All patients treated with this protocol will have radiographic progression of their tumor and be candidates for surgical resection of their recurrent lesion. After resection and intra-operative histologic confirmation of recurrence, catheters will be placed under direct visualization into the invaded tissue surrounding the resection cavity. After postoperative radiographic confirmation of catheter placement, drug infusion will proceed for 3 days. Toxicity will be evaluated over the subsequent 6 months, and tumor response will be assessed using serial MRI. There are theoretical benefits of convection enhanced delivery as a method for treating brain tumors with chemotherapy that have shown promise in laboratory studies. Direct delivery of chemotherapy into the brain has the potential to avoid many side effects of chemotherapy on other parts of the body such as the immune system, liver and kidney. This has proven true in laboratory studies as discussed below. Also, the blood vessels in the brain typically do not allow complex molecules such as chemotherapy to cross into the brain. Convection enhanced delivery circumvents this barrier and may allow for a much higher dose of chemotherapy to reach the tumor cells. Catheters for delivery of chemotherapy into the brain will be placed during your surgery for removal of the brain tumor. Chemotherapy will then be delivered for 72 hours after confirming the catheters are in the right place with a CT scan. This will begin approximately 24 hours after surgery. The patient’s medical team, including the neurosurgeon who performed the surgery, neuro-oncologist and nurses will monitor the infusion and the overall medical and neurologic status of the patient during the hospitalization. After finishing chemotherapy, the catheters will be removed. Catheter removal does not require another surgery – this will be done at the bedside as is routine for removal of these types of catheters.

This is a phase I study to evaluate the toxicity and safety of carboplatin administered by convection enhanced delivery into the tumor in patients with high grade glial neoplasms. These patients will have had primary therapy consisting of surgery for resection or biopsy, X-irradiation and concomitant administration of Temozolomide, followed by repetitive cycles of TMZ, and tumor recurrence that necessitates a second craniotomy. Additional data, such as radiographic tumor response and survival, will also be assessed. This information may provide the basis for future clinical work with convection enhanced delivery of carboplatin.

## 2.2 - Glioblastoma multiforme

Glioblastoma multiforme (grade IV astrocytoma, GBM) is the most common primary malignant brain tumor with an age-adjusted incidence of 3.05 per 100,000 per year in the United States[[7](#_ENREF_7)]. Despite multimodality treatment strategies that combine surgery, chemotherapy, and radiotherapy, high-grade gliomas are almost uniformly fatal[[8](#_ENREF_8)]. One of the most important advances in the treatment of glioblastoma multiforme (GBM) over the past 20 years has been the introduction of temozolomide in combination with photon irradiation, followed by repetitive cycles of temozolomide (TMZ)[[9](#_ENREF_9)]. Although a significant subset of patients were survivors at 24 months[[9](#_ENREF_9)], the overall increase in median lifespan of GBM patients was only 2.5 months (14.6 months vs. 12.1 months). Nevertheless, due to the size of the study, 573 patients, randomized into two arms, surgery + radiation-therapy (RT) or surgery + RT and concomitant TMZ, followed by repetitive cycles of TMZ, the difference in survival of the two groups was highly significant (p<0.001)[[9](#_ENREF_9), [10](#_ENREF_10)]. Five year follow-up revealed that the benefits of this therapy continued over the remaining 3 years of the study and the 5 year survival rate was 9.8% versus 1.9 % without TMZ[[11](#_ENREF_11)]. Although this approach rapidly has become the standard of care, it is clear that new innovative therapeutic approaches still are required, especially in patients with recurrent GBM.

## 2.3 - Carboplatin

Cisplatin and carboplatin are highly effective anti-cancer drugs that have been used to treat a wide variety of malignancies[[12](#_ENREF_12)]. The formation of platinum adducts with nucleophilic sites in DNA molecules causes cell cycle arrest in G_1_ and G_0_[[13](#_ENREF_13)], and the activation of apoptotic pathways[[14](#_ENREF_14)]. This can interfere with the repair of radiation-induced damage and may explain the interaction between platinated drugs (cisplatin and carboplatin) and ionizing radiation[[15-17](#_ENREF_15)]. Pre-clinical studies have shown that gliomas are sensitive to these agents. Phase II studies of intravenous carboplatin, administered as single agent or in combination with other therapeutics, however, have shown only a modest impact on outcome[[12](#_ENREF_12)]. Median progression-free and overall survival was approximately 3 and 9 months, respectively.

In studies carried out almost 30 years ago at The Ohio State University with the F98 rat glioma model, Kaneko et al.[[18](#_ENREF_18)] reported that systemic administration of cisplatin in combination with radiation therapy (RT) produced a significant prolongation in both median and mean survival times (56 d) and a 25% increase in life span (%ILS) of tumor bearing rats compared to radiation alone (44 d). Not long after, the European Organization for Research and Treatment of Cancer (EORTC) carried out a randomized clinical trial involving 285 patients to evaluate the effects of systemically administered cisplatin, with concomitant RT in patients with supratentorial malignant gliomas[[19](#_ENREF_19)]. This study failed to demonstrate any improvement in either progression free or overall survival times and it brought to an end any further clinical studies to investigate the combination of cisplatin and photon radiation to treat high grade gliomas. The clinical use of systemically administered platinum drugs to treat patients with brain tumors has been limited, not only by their systemic toxicity, but also by their poor ability to penetrate an intact blood-brain barrier (BBB), especially in areas where there are microinvasive deposits of tumor[[4](#_ENREF_4)]. Despite producing significant systemic toxicity, both the oral and intravenous routes of administration have not resulted in therapeutic concentrations of these drugs in the central nervous system (CNS). In order to optimize the activity of these agents for the treatment of malignant gliomas, alternative methods of drug delivery are needed.

## 2.4 - Convection Enhanced Delivery

Several approaches have been proposed to bypass the BBB and deliver anti-cancer drugs directly to the brain, thereby increasing tumor drug concentrations and reducing the associated systemic toxicity. These methods include intra-tumoral (i.t.) bolus injection[[20](#_ENREF_20)], surgical implantation of drug-loaded polymer matrices in the brain[[20-24](#_ENREF_20)] and convection enhanced delivery (CED) of drugs via catheters placed into the tumor and/or surrounding brain[[25](#_ENREF_25), [26](#_ENREF_26)]. CED is an innovative method to increase the uptake and distribution of low and high molecular weight therapeutic agents in the brain[[25](#_ENREF_25), [27](#_ENREF_27), [28](#_ENREF_28)]. Under normal physiological conditions, interstitial fluids move through the brain by both convection and diffusion. Diffusion of a drug in tissue depends upon its molecular weight, ionic charge and its concentration gradient within normal tissue and the tumor. The higher the molecular weight of the drug, the more positively charged the ionic species, and the lower its concentration, then the slower its diffusion. For example, diffusion of antibody into a tumor requires 3 days to diffuse 1mm from the point of origin. Unlike diffusion, however, convection or “bulk” flow results from a pressure gradient that is independent of the molecular weight of the substance. CED has been used to improve the targeting of both low and high molecular weight agents, including monoclonal antibodies (mAbs) and liposomes to the central nervous system (CNS). By applying a pressure gradient to establish bulk flow during interstitial infusion, the volume of distribution (V_d_) is a linear function of the volume of the infusate (V_i_)[[26](#_ENREF_26)]. CED has been used by one of us (RFB) to improve the uptake of Epidermal Growth Factor Receptor (EGFR) targeting mAbs and bioconjugates in rats bearing EGFR positive F98 gliomas[[29](#_ENREF_29), [30](#_ENREF_30)]. It has been used clinically by Sampson et al to improve the uptake of an EGFR targeted immunotoxin in patients with recurrent gliomas[[31](#_ENREF_31)]. It also has been used clinically by another member of our research team, Dr. E.A. Chiocca, to administer genetically engineered oncolytic Herpes simples virus (HSV) to patients with recurrent GBMs[[32](#_ENREF_32)]. Other studies describe use of convection-enhanced delivery of chemotherapy agents such as paclitaxel (Lidar, et al 2004) and (Mardor, et al 2001) in clinical trials for human malignant brain tumors. A recent publication (White, et al 2011) outlines the authors’ intent to perform a phase I clinical trial of carboplatin for recurrent malignant brain tumors. The protocol described in this article has many similarities to the protocol described here.

## 2.5 - Pre-clinical Studies of Intratumoral Administration of Carboplatin and Cisplatin

At least with chemosensitive tumors, there has been a clear relationship between the therapeutic response and increasing both the concentration and duration of exposure to carboplatin[[20](#_ENREF_20), [22](#_ENREF_22), [24](#_ENREF_24)] or cisplatin[[33](#_ENREF_33)]. Using the RG2 rat glioma model, with tumor cells implanted into the cortex, Emerich et al[[22](#_ENREF_22), [24](#_ENREF_24)] showed that sustained delivery of carboplatin with biodegradable microspheres, placed into the tumor, significantly improved survival compared to equipotent bolus injections[[24](#_ENREF_24)]. Similarly, Carson and his co-workers have shown that carboplatin, delivered by osmotic pumps into the brainstems of rats bearing F98 gliomas, significantly enhanced their survival[[16](#_ENREF_16), [34-37](#_ENREF_34)].

Using the F98 rat glioma model, Dr. Hélène Elleaume and her research team at the European Synchrotron Radiation Facility (ESRF) in Grenoble, France, have evaluated the therapeutic efficacy of either short-term intratumoral injection[[38](#_ENREF_38)] or CED of cisplatin[[39](#_ENREF_39)] or prolonged i.c. Alzet pump infusions or CED of carboplatin in combination with 6MV photon irradiation[[29](#_ENREF_29), [30](#_ENREF_30)] using a linear accelerator (LINAC). Kaplan-Meier survival plots are shown in Fig. 1. Untreated animals had a mean survival time (MST) of 23 ± 1 d., compared with 44 ± 3 d. for X-irradiated rats. Animals that had received carboplatin by Alzet pumps had a censored MST of 69 ± 20 d., with 3 rats surviving >195 d., at which time the study was terminated. Animals that had received carboplatin, by Alzet pumps followed by X‑irradiation, had a MST of >142± 21 d. with 6 of 11 rats (55%) alive at the end of the study[[29](#_ENREF_29)]. This was significantly different from irradiated animals (*p*=0.0014) or those that had received carboplatin alone (*p*=0.0472). The corresponding %ILSs, based on the median survival time, were 25%, and 85%, and 713% (censored) for carboplatin alone, radiotherapy alone, or chemoradiotherapy, respectively. It is noteworthy that in the chemoradiotherapy group, 8 of 11 rats (73%) were still alive at d. 100.

|  | Fig. 1. Kaplan-Meier survival curves for F98 glioma-bearing rats after chemoradiotherapy. Group 5: untreated animals (×); Group 6: X‑irradiation only (-); Group 7: carboplatin administered by ALZET^TM^ pump alone (▲), or Group 8: irradiation alone. In combination with X‑irradiation (△).The survival plots of all treatment groups were significantly different from the untreated controls (p<0.0015). The survival of the animals that received carboplatin, followed by a single 15 Gy dose of X-irradiation was significantly different from irradiated animals (p=0.0014) or those that received carboplatin alone (p=0.0472).^32^ |
| --- | --- |

These results are the best ever obtained with the F98 glioma model, which has been incurable by all other therapeutic modalities, except for boron neutron capture therapy (BNCT)[[40](#_ENREF_40)].

Dr. Barth and his research team also have carried out studies on the therapeutic efficacy of intracerebral delivery of carboplatin in combination with radiation therapy (RT) for treatment of the F98 rat glioma[[41](#_ENREF_41)]. Tumor cells were implanted stereotactically into the brains of syngeneic Fischer rats, and 13 or 17 d. later carboplatin was administered by either CED (20 µg/10 µL) over 30 min or by Alzet osmotic pumps (0.5 µg/µL/h for 168 h.) beginning at 7 d after tumor implantation. Using a Siemens LINAC rats were irradiated with a 15 Gy fractionated dose (5 Gy x 3) of 6 MV photons to the whole brain beginning on the day after drug administration. Other groups of rats received either carboplatin or X-irradiation alone. The tumor carboplatin concentration following CED of 20 µg in 10 µL was 10.4 µg/g, which was equal to that observed following i.v. administration of a lethal dose of 20 mg, which was 1,000 fold greater than the amount of drug administered by CED (20 µg versus 20,000 µg).

As shown in Table 1 and Fig 2A, rats bearing small tumors (20-25 mm^3^) treated with carboplatin and X-irradiation, had a MST of 83.4 d following CED and 111.8 d following Alzet pump delivery with 40% of the latter surviving >180 d (i.e. cured) compared to 55.2 d for CED and 77.2 d. for pump delivery of carboplatin alone and 31.8 d and 24.2 d, respectively, for X‑irradiated and untreated controls. There was no microscopic evidence of residual tumor in the brains of all long-term survivors. In contrast, as shown in Fib 2B, rats bearing large tumors (60‑80 mm^3^), the MST of untreated and X-irradiated animals were equivalent to those observed in rats bearing small tumors. However, rats bearing larger tumors that had received carboplatin by CED had a MST of 34.9± 8.2 d and those that received the combination treatment had a MST of 44.9±3.5 d. These results clearly demonstrated that tumor size was an important determinant of the response to therapy and animals with small tumors were more responsive than those with larger tumors. This underscores the importance of tumor resection, to reduce overall tumor burden, prior to administering carboplatin via CED, as described above.

It is noteworthy that oral administration or CED of TMZ with or without X-irradiation in F98 glioma bearing rats resulted in only a very modest increase in MSTs. X-irradiated rats had a MST 21.2± 0.8 d compared to MSTs of 17.0 ±1.2 d and 18.2±1.7 d, for TMZ administered either orally or by gavage, and 23.2 and 29.3±5.0 d for those that received it in combination with X‑irradiation. No statistically significant therapeutic gain was seen in rats that had received TMZ compared to X-irradiation alone.

**Table 1. Survival data of rats bearing small F98 gliomas following convection enhanced delivery of carboplatin with or without X-irradiation**

|  |  | Survival times (days) ^a^ | | |  | % Increased life span | |
| --- | --- | --- | --- | --- | --- | --- | --- |
| Treatment Group | N | Mean ± SE | Median | Range |  | Mean | Median |
| Carboplatin/CED + X‑irradiation^b^ | 18 | 83.4±13.1 | 58 | 37->180(4) |  | 245 | 152 |
| Carboplatin/CED | 18 | 55.2±7.8 | 46 | 35->180(1) |  | 128 | 100 |
| CarboplatinAlzet pump + X-irradiation | 5 | 111.8±31.5 | 78 | 53->180(2) |  | 362 | 239 |
| Carboplatin/Alzet pump^c^ | 6 | 77.2±23.0 | 59 | 44->180(1) |  | 219 | 156 |
| X-irradiation only | 5 | 31.8±1.2 | 32 | 28-33 |  | 31 | 39 |
| Untreated controls | 5 | 24.2±1.6 | 23 | 21-28 |  | 0 | 0 |

^a^Survival of >180 d indicates that these animals have been cured of their tumors. The number in parenthesis indicates the number of long-term survivors.

b Animals received 15 Gy of 6 MV LINAC X-rays, delivered in 5 Gy fractions on d 14, 15 and 16

c ALZET osmotic pump delivery carboplatin at rate 1 ll/h (84 lg) for 7 days (from d 7 to d 13)

**A**

**B**

Fig. 2. Kaplan-Meier survival plots of F98 glioma bearing rats following administration of carboplatin or by Alzet pumps alone or in combination with X-irradiation. Survival time in days after implantation have been plotted for untreated animals (⚫), X‑irradiation only (15 Gy) (⭘), CED of carboplatin (▼), CED of carboplatin (20 µg over 30 min) + X-irradiation (△), carboplatin, administered by Alzet pumps (84 µg g over 7 d) (■), carboplatin, administered by Alzet pumps + X-irradiation (□). B. Kaplan-Meier survival plots of rats F98 glioma bearing either small (~20-25 mm^3^) or large (~60-80 mm^3^) tumors. Survival times in days after implantation have been plotted for untreated controls (⚫), irradiated rats (⭘), CED of carboplatin in rats with small tumors (▼), CED of carboplatin + X-irradiation in rats with small tumors (△), CED of carboplatin in rats with large tumors (■), CED of carboplatin + X-irradiation in rats with large tumors (□).

The chemotherapy results in the F98 glioma model are in good agreement with those previously reported by Olivi *et al.*[[20](#_ENREF_20)] using carboplatin-loaded biodegradable polymers for treatment of this same tumor. They reported that 5% carboplatin-loaded polymer (~0.5 mg of drug), released over 8 to 13 days, beginning 5 days after tumor implantation, produced a threefold increase in median survival time (MeST) compared to controls. Systemic administration of carboplatin once per week for 3 weeks at a dose of 30 mg/kg/week also significantly prolonged survival of F98 glioma bearing rats, but this was less effective than intracerebral (i.c.) delivery by the carboplatin polymer. Therapeutic efficacy was dose-related with an increasing number of early deaths associated with escalating doses of the drug up to 0.5 mg. In our study, the amount of carboplatin administered by Alzet pumps was 84 μg and was well tolerated and resulted in 17% long-term survivors (>180 d) for carboplatin along and 40% when combined with RT[[41](#_ENREF_41)].

Carson *et al.* have reported that the infusion of carboplatin over a 7 day period with Alzet^TM^ pumps at a dose of 0.1 mg doubled the MST of Fischer rats bearing brainstem implants of either the F98 glioma or 9L gliosarcoma[[35](#_ENREF_35)]. Platinum concentrations in F98 glioma bearing rats were maximal in brain sections corresponding to the infusion site with diminished amounts (1 to 5 9µg/g tissue) in sections that were 3 mm from the point of infusion[[42](#_ENREF_42)]. However, this distribution pattern was observed in only half of the brains infused with rigid catheters such as those that we have used. In contrast, the use of flexible catheters resulted in the expected drug distribution in all of the rats, in both normal and tumor-bearing animals. It was suggested that rigid catheters might result in backflow of the drug into the subdural space along the catheter path[[42](#_ENREF_42)]. It is noteworthy that in this study the brainstem was not damaged by direct infusion of 200 µL of carboplatin (0.5 mg/mL) at a flow rate of 1 µL/h, and that there was no neurotoxicity until a lethal dose (dose ≥1.0 mg/mL) was administered[[38](#_ENREF_38)]. However, in a very recently reported study by the same group, up to 3 catheters were used to infuse carboplatin into the brainstems of non-tumor bearing Fischer rats[[34](#_ENREF_34)]. Using Alzet^TM^ osmotic pumps 0.1 mg of carboplatin or 5% dextrose was delivered via one, two or three cannulas at a flow rate of 1 µL/h over 7 days. Animals that received carboplatin showed some relevant neurological impairment during infusion compared to those that received 5% dextrose, and this persisted. It was concluded that carboplatin related neurotoxicity was important if the drug was infused into functional, highly eloquent structures of the brainstem[[43](#_ENREF_43)].

Degen et al.[[44](#_ENREF_44)] carried out a dose-escalation study of CED of carboplatin into the striatum of non-tumor bearing rats. No toxicity was observed with doses of 0.1 and 1.0 mg/mL, but occurred in rats that had received 10 µL of the highest dose of carboplatin (100 µg) with 2 of 4 of these animals dying. Tange et al.[[45](#_ENREF_45)] recently reported on the therapeutic efficacy of carboplatin (0.1 mg/mL) administered by means of Alzet osmotic pumps tumors (1 µL/h during 7 d.) to 9L gliosarcoma bearing rats. Serial MR imaging was carried out and this showed significantly smaller tumor volumes and an increased MST of 60 d. with a subset (3 of 8 rats) of long term surviving >100 d. These two studies are in concordance with our previously published data on i.c. delivery of carboplatin[[29](#_ENREF_29), [30](#_ENREF_30)], as well as those reported by Dr. Barth’s research team. Pertinent data relative to these studies are summarized in Table 2.

As reported by Strege *et al.*[[43](#_ENREF_43)], the Hopkins’ group also has carried out extensive studies in Cynomologus monkeys to establish the safety of carboplatin that has been infused into the brainstem over extended periods of time. Infusions at a flow rate of 0.42 µL/h were timed to deliver 0.025, 0.075, 0.25, and 0.75 mg/kg of carboplatin by day 30. There was no apparent toxicity in the group that received the lowest dose of carboplatin (0.025 mg/kg); the animals were clinically normal at day 30 when their accumulated dose reached ~80 µg. The animals in the second group (0.075 mg/kg) displayed neurotoxicity when their accumulated drug level reached 240-270 µg. Dose-dependent neurotoxicity only was observed only with doses >400 µg[[43](#_ENREF_43)]. The authors concluded that carboplatin can be chronically infused into monkey brainstems, but that neurotoxicity was the predominant side effect and that this was dose-dependent. Pertinent data relative to the primate studies are summarized in Table 3.

**Table 2. Selected Animal Studies Using Convection Enhanced Delivery for Intracerebral Administration of Carboplatin for Treatment of Brain Tumors**

| Site* | Species | Investigator | Catheters/  Cannulas | Volume | Infusion  Rate | Total Time | Dose | Comments |
| --- | --- | --- | --- | --- | --- | --- | --- | --- |
| H | Rat | Rousseau et al.[[30](#_ENREF_30), [46](#_ENREF_46)] | 1 | 20 or 40 µl | 30 µl/h | 40 or 80 min | 20 or 40 µg | Drug was given alone or with x-rays |
| H | Rat | Yang et al.[[41](#_ENREF_41)] | 1 | 10 µl | 20 µl/h | 30 min | 20 µg | Dose of 40 µg was neurotoxic |
| H | Rat | Degen et al.[[44](#_ENREF_44)] | 1 | 40 µl | 0.5 µl/h | 80 min | 40 µg | Toxicity at 100 µg |
| BS | Rat | Wu et al.[[37](#_ENREF_37)]  Thomale et al.[[34](#_ENREF_34)] | 1 - 3 | 168 µl | 1 µl/h | 7 d | 100 µg | No persisting neurological deficits. |
| BS | Primate | Carey et al.[[47](#_ENREF_47)] | 1 | 302.4 ml | 0.42 µl/h | 30 d | 2.6 µg/d – 90 µg/d | Maximum “no clinical event” dose was 170 µg total |
| BS | Primate | Strege et al.[[43](#_ENREF_43)] | 1 | 141.1 ml | 0.42 µl/h | 30 d | 200 µg | Maximum dose that produced no neurologic symptoms |

*H designates cerebral hemisphere; BS designates brain stem

**Table 3. Selected Human Studies Using Convection Enhanced Delivery for Intracerebral Administration of Therapeutic Agents for Treatment of Brain Tumors**

| Therapeutic Agent | Site* | Investigator | Catheters | Volume | Infusion  Rate | Total Time | Dose | Comments |
| --- | --- | --- | --- | --- | --- | --- | --- | --- |
| IL13-PE38QQR IT^¶^ | H | Kunwar et al.[[48](#_ENREF_48), [49](#_ENREF_49)] | 2-4 | 72 ml | 750 µl/hr | 96 h | 48 µg | Study closed |
| Reolysin** | H | Aghi et al.[[32](#_ENREF_32)] | 4 | 28.8 ml | 400 µl/hr | 72 h |  | Study closed |
| EGFR IT^‡^ | H | Sampson et al.[[31](#_ENREF_31)] | 2-4 | 72 ml | 750 µl/hr | 96 h |  | Also infused I-labeled albumin and Gd |
| Carboplatin | BS | Jallo/Guarnieri et al.^†^ | 1 | 12 µl | 0.5 µl/hr | 24 h |  | Proposed but not done |
| Carboplatin | H | Elder/Cavaliere/Barth (IND) | 2-4 | 54 ml | 750 µl/hr | 72 h | 100 µg – 10 mg | Our study |

*H designates cerebral hemisphere; BS designates brain stem. ^‡^This was an EGFR targeting immunotoxin (IT).

^¶^This was an IL-13 receptor targeting immunotoxin. ^†^Pre-IND #71,156, Intratumoral infusion of paraplatin for diffuse brainstem glioma.

**This was an oncolytic herpes virus.

## 2.6 - Human Studies of Intracerebral Administration of Platinum Agents

A variety of drug delivery systems has been proposed for the treatment of patients with malignant gliomas to deliver anti-cancer drugs locally in a sustained slow-release manner [[20](#_ENREF_20), [21](#_ENREF_21), [33](#_ENREF_33), [50](#_ENREF_50)]. To the best of our knowledge, there are only two clinical reports describing the direct i.c. infusion of platinated drugs for glioma treatment. Bouvier et al.[[26](#_ENREF_26)] infused 8.2 mg of cisplatin over 10 days into the left frontoparietal lobe of a patient with a recurrent malignant glioma using 68 catheters in clusters of four, each catheter connected to an Alzet^TM^ pump (121 µg of cisplatin per catheter). There were no adverse side effects associated with catheter implantation, chemotherapy, or catheter removal. Although chemotherapy halted progression of the tumor, it recurred, and the patient died 6 months after treatment[[26](#_ENREF_26)]. The only evidence of tissue damage was an infiltrate of foamy macrophages and foreign body giant cells that were reacting to microscopic fragments of bone. Contiguous brain showed gliosis, but no evidence of hemorrhage. Although this was not really CED, it resulted in higher drug concentrations than otherwise would have been obtainable by systemic administration, and provides one data point demonstrating that direct infusion of platinated drugs can be performed safely.

In the other study involving 17 patients with glioblastoma (GBM), Sheleg et al.[[23](#_ENREF_23)] administered cisplatin, incorporated into biodegradable polymers into the cavity created following subtotal resection of the tumor. Twenty polymer discs (1.5 x 1.5 cm^2^), each loaded with 1 mg/cm^2^ of cisplatin, were placed in the cavity created following resection of the tumor. Two weeks after surgery, the patients received radiotherapy (2 Gy per fraction for a total dose of 60 Gy). An additional 21 patients with GBM only received radiotherapy. As shown by brain CT, it was determined that complete biodegradation of the cisplatin containing discs had occurred by 4-5 weeks following their placement in the tumor bed. The combined treatment significantly increased (p=0.00001) the median survival time (427 d.) compared to that observed in GBM patients who had received radiotherapy alone (211 days). Although, there was no autopsy data to indicate what, if any, neuropathologic changes had occurred in the treated patients, clinically the treatment seemed to have been well-tolerated.

# 3 - Pharmaceutical Information

The carboplatin infusion will be prepared by the OSUMC / James Pharmacy Investigational Drug Service. Carboplatin will be received from their pharmacy wholesaler as a sterile, lyophilized powder which is stable at room temperature until the expiration date on the bottle. OSUMC / James Pharmacy will dissolve the carboplatin in 0.9% sodium chloride using aseptic techniques to the concentration desired based on the cohort level. The final infusate will be an isotonic solution received in syringes which is stable for 24 hours.

Carboplatin [diammine(1,1-cyclobutanedicarboxylato-(2)-0,0’) platinum, NSC 241240, IND 19,314] (CBDCA). It is a cyclobutane dicarboxylate derivative of the potent antitumor platinum II compound, DCCP. Several studies into the mechanism of action of platinum II compounds have compared the biochemical reactivities of *cis* and *trans* stereoisomers as the latter have virtually no antitumor activity; differences in reactivities therefore help elucidate the mechanism(s) of activity of the *cis* compounds. Both *cis* and *trans* compounds form inter-(DNA-DNA, DNA-protein) and intrastrand crosslinks, and both react with phosphate but not sugar groups of DNA. Only *cis* compounds, however, show preferential reactivity with G-C base pairs. The primary lesion formed with bidentate platinum compounds such as CDDP and CBDCA, as a result of this reaction, is an intrastrand cross-link between two adjacent guanines; interstrand crosslinks are relatively rare. Physically, the association with both CDDP and CBDCA with DNA has been found to induce a “premelting” effect in intact DNA, characterized by a weakening or rupture of hydrogen bonding between bases and resulting in an opening of the double helix. A DNA shortening effect has also been seen. In contrast, *trans* compounds cause the formation of single stranded, denatured DNA. Carboplatin is a highly effective anti-cancer drug that have been used clinically to treat a variety of malignancies[[12](#_ENREF_12)]. The formation of platinum adducts with DNA can interfere with the repair of radiation-induced damage, which could explain the synergistic interaction between either cisplatin or carboplatin and ionizing radiation, which have been observed in the F98 rat glioma model[[16](#_ENREF_16), [35](#_ENREF_35), [36](#_ENREF_36)].

# 4 - Patient Eligibility Criteria

4.1 - Population:

Patients, aged 18 to 70, with grade III or IV astrocytoma (anaplastic astrocytoma or glioblastoma), or grade III oligodendroglioma or oligastrocytoma (anaplastic oligodendroglioma or anaplastic oligoastrocytoma) at first or second recurrence are eligible to enroll in this protocol. Subjects are required to have radiographic confirmation of disease progression and a Karnofsky Performance Status of at least 60. Eligible patients must be at least 6 weeks out from last dose of nitrosoureas, bevacizumab and other antiangiogenic therapies, 8 weeks out from completion of radiotherapy (fractionated or stereotactic), and 4 weeks out from last dose of other cytotoxic chemotherapies. Patients cannot be receiving other investigational agents and must be on a stable dose of dexamethasone for ≥1 week prior to baseline MRI. The tumor progression must be amenable to craniotomy for resection. Candidates for the study include only those patients for whom craniotomy for tumor resection is recommended as treatment for their tumor progression.

## 4.2 - Inclusion Criteria

- All patients must have progressive disease for which craniotomy and tumor resection is recommended as treatment.
- All patients must sign a consent form indicating that they are aware of the investigational nature of the study. The informed consent form will indicate that the patient has been made aware of all other appropriate therapies.
- Patients with histologically confirmed grade III or IV astrocytoma, oligoastrocytoma, and oligodendroglioma who are at first or second recurrence
- All patients require an initial diagnosis of a malignant glioma as outlined in the inclusion criteria which must be confirmed at the treating facility.
- Patients must have unequivocal evidence of tumor progression by MRI performed no longer than 28 days prior to study registration.
- Patients must have pathologically confirmed recurrence at the time of catheter placement.
- Patients must be on a stable or decreasing dexamethasone dosage for at least 1 week prior to baseline MRI.
- Patients must have been treated previously with radiation therapy and treatment must have been completed at least 8 weeks prior to surgery for catheter implantation
- Last dose of cytotoxic chemotherapy must have been at least 4 weeks (6 weeks for nitrosoureas) prior to catheter placement. Patients are eligible if they received bevacizumab or other anti-VEGF therapies, although the most recent dose must be at least 6 weeks prior to catheter placement.
- Patients previously treated with stereotactic radiosurgery, stereotactic radiotherapy, brachytherapy, Gliadel wafers or other intratumoral chemotherapy are eligible.
- Patients must have recovered from all prior therapy.
- Age ≥ 18 and ≤70 years of age
- Patients must have a life expectancy of ≥ 3 months and a Karnofsky performance status ≥ 60.
- Patients must have normal organ and marrow function as defined below:
  - leukocytes >3,000/mcL
  - absolute neutrophil count >1,500/mcL
  - platelets >100,000/mcL
  - hemoglobin >9 g/dL
  - serum calcium <12.0 mg/dL
  - total serum bilirubin < institutional ULN
  - AST(SGOT)/ALT(SGPT) ≤2.5 X institutional ULN
  - creatinine <1.5 X institutional ULN
- Women of child bearing years must have a negative pregnancy test (serum or urine) within 1 week of study entry. Men and women of reproductive potential must agree to use an effective contraceptive method including one of the following: surgical sterilization (tubal ligation for women or vasectomy for men); approved hormonal contraceptives (such as birth control pills, Depo-Provera or Lupron Depro); barrier methods (such as condom or diaphragm) used with a spermicide cream or an intrauterine device (IUD).
- Patient or designated individuals with durable medical power of attorney must give written informed consent prior to any study-specific procedures being implemented.
- Both men and women and members of all races and ethnic groups are eligible for this trial.

## 4.3 - Exclusion criteria

- Patients with infratentorial, multifocal, or pathologically confirmed CSF disseminated tumor.
- Patients that have been treated with > 3 prior chemotherapy regimens
- Pregnant or lactating women or women of childbearing potential and men who are sexually active and not willing/able to use medically acceptable forms of contraception.
- Patients who have a history of bleeding disorders including congenital or acquired coagulopathies
- Known AIDS based upon current CDC definition or other acquired or congenital disorder of the immune system.
- Patients with unstable or serious concurrent illness including, but not limited to, ongoing or active infections requiring IV antibiotics or psychiatric illness/social situations that would limit compliance with study requirements are ineligible. (If patient has a stable chronic infection requiring oral antibiotics, the patient may be treated at the investigators discretion; however a clinical note must include the justification regarding the safety of treating the patient)
- Patients who have received any other investigational agent in a 28-day period prior to enrollment in this study.
- Patients whose tumors are located less than 2 cm from the ventricles.
- Patients taking greater than 12mg daily of dexamethasone.
- Prior invasive malignancy that is not low-grade glioma, glioblastoma or gliosarcoma (except non-melanomatous skin cancer or carcinoma in situ of the cervix) unless the patient has been disease free and off therapy for that disease for a minimum of 3 years

# 5 - Treatment Plan

5.1 - Study Design:

Phase I dose escalation study

5.2 - Proposed trial:

Carboplatin in a volume of 54 ml will be administered intracerebrally by convection enhanced delivery at a flow rate of 0.75 ml/hr over 72 hr. The first patient will receive an initial starting dose of 2 µg over 72 hrs (0.667 µg/24 hr). This first patient will be observed for 28 days after completion of the infusion, prior to enrollment of additional patients. In the absence of dose-limiting toxicity (DLT), 3 more patients will be entered at the next higher dose level, which will be double the prior dose (i.e., 4 µg over 72 hrs). If 1of 3 patients exhibits DLT, an additional 3 patients will be entered at that dose level. Dose escalation will continue until DLT occurs in at least 2 out of 6 patients. The maximum tolerated does (MTD) will be defined as the highest dose level at which 3/3 or 5/6 patients could be treated without DLT. Dosage escalation will proceed in subsequent cohorts of 8 µg, 16 µg, 32 µg, 64 µg, 85 µg, 107 µg and 128 µg. If the MTD has not been reached, we will submit a request to the FDA with a schedule for continued escalation until DLT is reached. If 2 of 3 patients demonstrate DLT at 2 µg, the dose will be reduced to 1 µg for the next three patients. DLTs are defined, in the presence of supportive care, as any Grade 3 or 4 toxicity that is possibly, probably, or definitely related to the study drug. The maximum tolerated dosage is defined as the dose level below that causing DLT in at least two patients. The length of the DLT window will be at least 28 days, and this time interval must elapse before completion of one cohort and initiation of the next.

5.3 - Population:

Patients, aged 18 to 70, with grade III or IV astrocytoma (anaplastic astrocytoma or glioblastoma), or grade III oligodendroglioma or oligastrocytoma (anaplastic oligodendroglioma or anaplastic oligoastrocytoma) at first or second recurrence are eligible to enroll in this protocol. Subjects are required to have radiographic confirmation of disease progression and a Karnofsky Performance Status of at least 60. Eligible patients must be at least 6 weeks out from last dose of nitrosoureas, bevacizumab and other antiangiogenic therapies, 8 weeks out from completion of radiotherapy (fractionated or stereotactic), and 4 weeks out from last dose of other cytotoxic chemotherapies. Patients cannot be receiving other investigational agents and must be on a stable dose of dexamethasone for ≥1 week prior to baseline MRI. The tumor progression must be amenable to craniotomy for resection. Candidates for the study include only those patients for whom craniotomy for tumor resection is recommended as treatment for their tumor progression.

5.4 - Outline of Treatment Plan – details below the outline

- Pre-operative assessment for enrollment in clinical trial (see above)
  - Tumor board confirms that the patient is an appropriate candidate for the clinical trial based on diagnosis and radiographic evidence of recurrent disease
    - Inclusion criteria met (see list above)
    - Exclusion criteria met (see list above)
  - The clinical trial is fully explained to the patient including all risks of the clinical trial and informed consent is obtained
- Surgery
  - Surgery occurs in the operating room at OSU Main Hospital
  - There are multiple objectives of surgery:
    - The tumor is surgically removed
    - Intraoperative frozen section confirms high grade glioma
    - Infusion catheters (2-4) are inserted into the brain around the tumor resection cavity
- Post-operative admission – hospitalization after surgery occurs in the OSU Main Hospital
  - patient is admitted to the ICU for the first 24 hours after surgery during which time the following occurs:
    - Monitoring for adverse events occurs continuously during hospitalization
    - vital signs and neurologic assessment hourly
    - CT to assess catheter placement
    - Labs as per study calendar (see below)
    - Neurologic assessment by neurosurgeon (Elder) and resident house staff daily
    - Neurologic monitoring by RN as per ICU standard of care
    - Once the CT scan has confirmed catheter placement, carboplatin infusion begins
      - management of carboplatin infusion by surgical team as directed by attending neurosurgeon (Elder)
      - Carboplatin infusate is supplied in a syringe at a concentration determined by the dosing algorithm described above
      - Syringe placed in infusion pump that controls infusion rate
      - Syringe connected to infusion catheters via infusion tubing
      - Each syringe contains 24 hours of infusate
      - Syringe is changed at 24 and 48 hours
      - Total infusion time is 72 hours
  - After 24 hours in the ICU, the patient is transferred to the ward in the James if the patient is stable for transfer to the ward
    - Carboplatin infusion continues for a total of 72 hours
    - Daily neurologic assessment by neurosurgeon (Elder) and resident house staff
    - Routine neurologic and vital signs assessment by RN as per standard of care and at least every 4 hours
    - Daily labs as per study protocol (see below)
  - Catheter removal at bedside after infusion completed – catheters are removed in a similar fashion as standard ventricular catheters. A member of the neurosurgical team as directed by the neurosurgical attending will remove each catheter and place a stitch in the scalp at the exit point of the catheter
  - An MRI to assess for residual tumor will be performed after the catheters are removed
  - Discharge from hospital as per standard of care – discharge criteria after brain tumor resection will be met prior to discharge home
- After discharge home
  - Serial imaging, labs and neurologic assessments as per study calendar (see below)

## 5.5 - Pre-operative safety

All patients enrolled in the trial will have been discussed at the weekly neuro-oncology tumor boards and deemed appropriate for the study based on eligibility and exclusion criteria (see section 4). All enrolled patients must sign the surgical consent and study consent.

5.6 - Surgery:

Patients will undergo surgery, which includes tumor resection and catheter placement, in the operating room. On the day of tumor resection, up to 4 barium-impregnated catheters (Medtronic Becker EDMS Ventricular Catheter 20cm Barium Impregnated cerebrospinal fluid ventricular catheters; Medtronic, Inc., Minneapolis, MN or equivalent) will be placed stereotactically into gadolinium-enhancing areas and/or in areas of FLAIR/T2 abnormalities. Prior use of ventricular catheters for intracerebral convection enhanced delivery has been approved by the U.S. Food and Drug Administration (BB-IND-9184) and published in peer-reviewed literature[[31](#_ENREF_31)]. Catheters can be placed stereotactically into infiltrated tumor areas unlikely to be resected or catheters can be advanced from the tumor cavity into surrounding brain. If there is no histologic confirmation of a malignant glioma via frozen section during tumor resection, for example in a case where the recurrent lesion actually represents another histologic entity such as radiation necrosis, catheters will not be placed.

The surgical procedure which is conducted in an operating room at OSU Main Hospital will include standard craniotomy steps such as skin incision, removal of part of the skull, opening the dura, identifying and resection the recurrent brain tumor and achieving hemostasis. At this point during standard tumor resection, the tissues would then be closed and the surgery would conclude. This would include closing the dura, affixing the bone flap to the skull with titanium plates and suturing the overlying soft tissues back together. Instead, prior to closing the dura, two to four infusion catheters will be placed into the brain surrounding the resection cavity. Each catheter will then be tunneled under the scalp and through the skin such that the end of the catheter is outside of the head and accessible after surgery. A luer lock cap will be secured to the catheter such that it can subsequently be connected to the infusion system. The dura is sutured together and bone flap reaffixed to the skull in the standard manner, with the only difference being that the catheters traverse these tissues. The catheters pass throught the skin at a different site than the skin incision. This procedure by which catheters are brought from the intracranial cavity to outside of the head is identical to that used for placement of a variety of cranial catheters such as intraoperative placement of ventriculostomy catheters and subdural drains, both of which are common neurosurgical procedures.

5.7 - Carboplatin infusion:

Patients enrolled in the study will undergo craniotomy for tumor resection. During this surgery, at the conclusion of tumor resection, they will have stereotactic insertion of up to 4 catheters into the area surrounding the resection cavity. Catheter placement will be performed by the neurosurgeon performing tumor resection. Catheter placement will occur during the same surgery as the tumor resection; a second surgery for catheter placement is not required. Carboplatin infusion will commence after postoperative CT confirmation of catheter placement. . Carboplatin infusion will not occur in the operating room. Infusion will proceed for 72 hours at which point the catheters will be removed at the bedside. The patient will be monitored in the hospital for an additional 12-24 hours prior to discharge.

5.8 - Assessments:

CT within the first 12 hours after surgery will verify catheter placement. MRI will be performed after catheter removal to assess, if possible, the volume of distribution of the infusate. While the patient is in the hospital, clinical evaluation will be performed per standard neurological-critical care guidelines. Carboplatin infusion and patient monitoring during the hospitalization will be conducted by the patient’s medical team including the neurosurgeon who performed the procedure, neuro-oncologist, nursing team and associated house staff. Thereafter, study patients will be evaluated clinically every 4 weeks for the first 16 weeks and every 8 weeks until 30 weeks. Evaluation will include full neurological and general examination, mini-mental status examination, and Karnofsky Performance Status. Serum carboplatin levels will not be assessed. MRI, performed every 2 months, will be evaluated per McDonald criteria. Additional ancillary imaging will be used when imaging changes are suggestive of a treatment related effect, or if the clinical scenario requires. Toxicity will be assessed according to the National Cancer Institute Common Terminology Criteria for Adverse Events, Version 4.0.

5.9 - Postoperative care and carboplatin infusion:

Immediate postoperative management will occur in the hospital and will follow the routine standard of the neurosurgeon and the neuro-oncology team. Vital signs and neurologic status will be assessed by nursing as per standard ICU protocol and recorded hourly for the first 24 hours after surgery and then at least every 8 hours for the remainder of hospitalization. Daily labs as per the study calendar will be assessed (see section 7). Infusion of carboplatin will occur in the hospital, primarily in the James Hospital, and will be initiated after CT confirmation of catheter location.

A CT will be obtained a maximum of 12 hours before the start of the infusion to confirm catheter position. Infusions will start preferably on postoperative day 1, but could be delayed up to postoperative day 7, based on patient’s condition. If the patient’s condition does not allow for infusion, the catheters will be removed. Once catheter placement has been confirmed, patients will undergo infusion of carboplatin. Carboplatin will be infused at a rate of 0.75 mL per hour over 72 hours via a syringe pump. The total flow rate (0.75 ml/hr will be divided among the catheters placed. The catheters and syringe pumps will be attached to a bedside programmable syringe-type microinfusion pump (Medex/Medfusion 3500 Syringe pump or equivalent, which are immediately available for use) via infusion tubing (Vygon Neuro Infusor Infusion catheter system).

The infusion pump will be monitored by the surgical team, including attending neurosurgeons, resident house staff and nurse practitioners. Decisions regarding initiation of infusion and determining if there are problems with the infusion will be the responsibility of the surgical team and supervised by one of the neurosurgical attending physicians (Elder). The infusate will be prepared and supplied by The OSUMC / James Pharmacy Investigational Drug Service (see section 3) at the dose as determined by the dosing schedule (see page 18, section entitled ‘dosing paradigm’) and will be changed every 24 hours by the surgical team. A syringe-type microinfusion pump (see paragraph above) will control the rate of carboplatin infusion into the infusion catheter. The syringe will connect to the infusion catheters via infusion tubing. The infusion rate will be 0.75 ml/hr. Nursing staff will record that the infusion is running, but will otherwise have no responsibilities regarding the infusion pump. For any problems with the infusion or if the infusion pump alarms, the nurses will notify the neurosurgical team. During infusion, vital signs and neurologic status will be assessed and recorded at least every 4 hours. A specific person designated for monitoring the infusion pump and this person’s contact information will be clearly identified in the chart and provided to the patient’s nurse. This person will be a member of the neurosurgical team and will be Dr. Elder, Dr. Cavaliere or Dr. Newton or a physician or nurse directly supervised by Dr. Elder, Dr. Newton or Dr. Cavaliere.

## 5.10 - Inpatient care

All patients will be admitted to the neurosurgical intensive care unit (NICU) after surgery. They will receive standard postoperative care including hourly vital sign and neurologic assessment. Patients will be transferred from the NICU to the stepdown unit or ward based on their clinical status. They will remain in a monitored setting (ICU, stepdown unit or ward) for the duration of their infusion, receiving continuous vital sign monitoring and frequent medical and neurologic examinations. Once the catheters have been removed, they will receive standard postoperative care provided to patients who have had craniotomy for brain tumor resection.

The admitting neurosurgeon and house staff will be responsible for documenting daily neurologic and medical examinations and responding to any acute changes in neurologic or medical status. A separate NICU attending and house staff team will provide additional examinations while the patient is in the NICU. Daily blood tests will monitor for marrow suppression, electrolyte imbalances and liver dysfunction.

CT within the first 12 hours after surgery will verify catheter placement and assess for intracranial hemorrhage. MRI will be performed after catheter removal to assess for residual enhancement and, if possible, the volume of distribution of the infusate. While the patient is in the hospital, clinical evaluation will be performed per standard neurological-critical care guidelines. Baseline examinations prior to surgery and 24 hours after surgery will include full neurological and general examinations, mini-mental status examination and Karnofsky Performance Status.

Neurologic status will be monitored as per standard of care for postoperative brain tumor resection patients and is described in sections 5 and 7. Daily examinations will be conducted by the surgeon (Elder, Chiocca) and house staff (residents). Serial vital signs and neurologic assessments by nursing staff will occur as per standard of care, including hourly assessments in the ICU during the first 24 hours after surgery and assessments every 4-8 hours thereafter. This may be adjusted based on the clinical/neurologic status of the patient. Neurologic examinations include an assessment of mental status including level of arousal and orientation, cranial nerve examination and motor/sensory examination of the extremities. Of note, the toxicities in primate studies referenced in the protocol were in a protocol for carboplatin infusion into the brain stem. We will not be performing infusion into the brain stem or any other eloquent structure as per the protocol.

5.11 - Post-discharge care / Outpatient monitoring:

Post-operative outpatient visits, laboratory tests and imaging will be in the James clinic and OSU outpatient testing facilities and will adhere to the calendar provided in the section 7.

After discharge, study patients will be evaluated clinically every 4 weeks for the first 16 weeks and every 8 weeks thereafter until 30 weeks. Evaluation will include full neurological and general examination, mini-mental status examination, and Karnofsky Performance Status. MRI, performed every 8 weeks, will be evaluated per McDonald criteria by a neuroradiologist (see below). Additional ancillary imaging will be used when imaging changes are suggestive of a treatment related effect, or if the clinical scenario requires. Toxicity will be assessed according to the National Cancer Institute Common Terminology Criteria for Adverse Events, Version 4.0

## 5.12 - Dosage escalation plan

The starting dose of carboplatin will be 2 µg. The carboplatin dose will be escalated in cohorts of 3 (maximum of 6 patients in each dose level if DLT occurs). Three patients will be entered first at Dose Level 1. In the absence of DLT, 3 more patients will be entered at the next higher dose level. If 1of 3 patients had DLT, an additional 3 patients will be entered at that dose level. Dose escalation will continue until DLT occurs in at least 2 out of 6 patients. The MTD will be defined as the highest dose level at which at least 5/6 patients could be treated without DLT. If 2 of 3 patients in Dose Level 1 demonstrate DLT, the next cohort of patients will receive Dose Level 0. At least 28 days must elapse between completing enrollment in a cohort level and beginning treatment at the next cohort level.

### 5.12.1 - Dose escalation schedule

The Dose Escalation Schedule is indicated in the following table. Carboplatin will be administered in a volume of 54 mL at a flow rate of 0.75 mL/hr over 72 hrs.

| Dose Escalation Schedule | |
| --- | --- |
| Dose Level | Carboplatin Dosage |
| Level 0 | 1 µg |
| Level 1 | 2 µg |
| Level 2 | 4 µg |
| Level 3 | 8 µg |
| Level 4 | 16 µg |
| Level 5 | 32 µg |
| Level 6 | 64 µg |
| Level 7 | 85 µg |
| Level 8 | 107 µg |
| Level 9 | 128 µg |

### 5.12.2 – Dose escalation Justification

Justification for these doses is as follows:

In rats, we routinely have administered carboplatin by CED over 30 min to one cerebral hemisphere (600 mg), and this was well tolerated. The rat brain weighs ~1.2 g or 600 mg per cerebral hemisphere. The volume administered was 10 µL containing 20 µg of carboplatin, which was equivalent to 2 µg of carboplatin per 60 mg of rat brain. This was equivalent to 0.033 µg of carboplatin in 0.0165 µL/mg brain or 33 µg in 16.5 µL/g brain. The human brain is 1000 times larger at 1.2 kg total and 600 g per hemisphere. If we scale up the rat dose to an adult human brain, this would be equivalent to 19.9 mg in 9.9 mL per cerebral hemisphere (33 µg/gm wt brain x 600=19,800 µg in 9900 µL=19.8 mg of carboplatin in 9.9 mL), which is 156X higher than the highest dose of carboplatin that we propose to administer (128 µg in 54 mL). The relative volume being infused as a function of time is much less. For the rat, 10 µL was infused into a 600 mg hemisphere over 0.5 hours resulting in a flow rate of 33 µL/g/hour. In our proposal, 54 ml infused into a 600 g hemisphere over 72 hours yields a flow rate of 1.25 µL/g/hour. Furthermore, the concentration of carboplatin in the rat experiments was 2 mg/ml, whereas the highest concentration of carboplatin in our proposal is 2.37 µg/ml.

In Strege, Jallo and Guarnieri’s report[[43](#_ENREF_43)], they stated that the MTD of carboplatin delivered to the brain stem of a 200 g Fischer rat by Alzet pumps at a flow rate of 1 µL/h over 7 days was 100 µg. They used a conversion factor of 0.5 to estimate the MTD to a 3 kg monkey, which was estimated to be 0.25 mg/kg (3 mg/m^2^) of carboplatin. Based on this, doses of carboplatin were diluted to be delivered over more than 30 days, and were delivered at 0.42 µL/h. Two animals received 0.1 x eMTD (0.025 mg/kg); three received 0.3 x eMTD (0.075 mg/kg); five received 1 x eMTD (0.25 mg/kg); and three received 3 x eMTD (0.75 mg/kg.)

Permission was requested and received (please see letter in the Appendix) to reference IND #71,156 submitted by George Jallo, MD from Johns Hopkins University. This proposed IND contains toxicity data on the administration of carboplatin by CED to the brainstem of primates, as well as safety data regarding the use of catheters implanted into neural tissue. This proposed protocol was designed to treat a pediatric population with brainstem tumors and the dosing regimen that was recommended to them by the FDA was based on this. Tables 2 and 3 provide further references used for selecting the total dose of carboplatin as well as the variables chosen for infusion.

Based on these considerations, we believe that the Dosing Escalation Schedule indicated in the above table represents a safe starting point. If it turns out that the 128 µg dose is well tolerated, then permission from the FDA will be obtained to further escalate the dose of carboplatin until toxicity has been demonstrated.

## 5.13 - Dose-Limiting Toxicity (DLT)

DLTs are defined, in the presence of supportive care, as any Grade 3 or 4 toxicity that is possibly, probably, or definitely related to the study drug. The maximum tolerated dosage is defined as the dose level below that causing DLT in at least two patients. The length of the DLT window will be at least 28 days, and this time interval must elapse before completion of one cohort and initiation of the next.

Non-hematologic toxicities such as rash, nausea, vomiting and hypertension will only be considered DLTs if they remain grade 3 or greater despite maximal medical therapy 72 hours after maximal medical therapy is initiated. The first deep venous thrombosis or pulmonary embolism will not be considered a DLT because these complications are common among patients with high-grade gliomas. Patients who develop DVT and/or PE may receive anticoagulation therapy and resume the clinical trial once clinically stable. A second DVT or PE on optimal medical therapy will be considered a DLT.

| **Number of Patients with DLT**  **At a Given Dose Level** | **Escalation Decision Rule** |
| --- | --- |
| 0 out of 3 | Enter 3 patients at the next dose level |
| > 2 | Dose escalation will be stopped. This dose level will be declared the maximally administered dose (highest dose administered). Three (3) additional patients will be entered at the next lowest dose level if only 3 patients were treated previously at that dose. |
| 1 out of 3 | Enter at least 3 more patients at this dose level.   - If 0 of these 3 patients experience DLT, proceed to the next dose level. - If 1 or more of this group suffer DLT, then dose escalation is stopped, and this dose is declared the maximally administered dose. Three (3) additional patients will be entered at the next lowest dose level if only 3 patients were treated previously at that dose. |
| <1 out of 6 at highest dose level below the maximally administered dose | This is generally the recommended phase 2 dose. At least 6 patients must be entered at the recommended phase 2 dose. |

The table above describes the dose escalation algorithm. If a DLT is encountered in 1 patient at a given dose level/cohort, an additional 3 patients will be enrolled at that dose level. If 2 patients in any cohort experience a DLT than the next lower dose level will be considered the MTD and no further dosage escalation will be performed. Three additional patients will be treated at the next lowest dose level if only 3 patients were previously treated at that level. If 3 patients are treated at the maximum dose without experiencing a DLT, an additional 3 patients will be treated at that dose level. At least 6 patients must be treated at the MTD. At least 28 days must elapse between the completion of one cohort to the beginning of the next cohort.

## 5.14 - Criteria for discontinuation of treatment

- DLT as defined by grade 3 or 4 toxicity according to the National Cancer Institute Common Terminology Criteria for Adverse Events, version 4.0.
- Grade 2 intracranial hemorrhage
- Request by the patient for discontinuation.

# 6 - Regulatory and Reporting Requirements

## 6.1 - Adverse Events (AE)

### 6.1.1 - Adverse Event Definition

An adverse event is an untoward medical event in a patient which does not necessarily need to have a casual relationship with the treatment. AEs will be reported according to the NCI Common Terminology Criteria for Adverse events (CTCAE) and Common Toxicity Criteria (CTC) version 4.0.

### 6.1.2 - Attribution of an AE

The relationship of an AE to the study medication will be established:

- Definite – AE Clearly related to the study treatment
- Probable – AE likely related to the study treatment
- Possible – AE may be related to the study treatment
- Unlikely – AE doubtfully related to the study treatment
- Unrelated – AE clearly not related to study treatment

## 6.2 - Serious Adverse Event Reporting (SAE)

An SAE is an adverse event that is:

- Life threatening
- An important medical event in the opinion of the principal investigator
- Results in:
  - Death
  - In-patient hospitalization or prolonged existing hospitalization
  - Persistent or significant disability or incapacity
  - Congenital anomaly/birth defect
  - Or in the opinion of the investigator represents significant hazard or potentially serious harm to subjects or others

### 6.2.1 - Non-serious adverse event definition

An adverse event is any untoward medical occurrence that does not meet the definition of serious (section 6.2). These do not have to be reported via expedited reported mechanism unless the treating physician feels it necessary. All AEs (including SAEs) must be reported in routine case report forms.

## 6.3 - Unexpected Adverse Event

An unexpected adverse event is any event where the nature and the severity of the event is not consistent with the previously reported documentation including the Investigator’s Brochure.

## 6.4 - AE Reporting

Adverse events which are unexpected and attributed to the investigational product must be reported to the Internal Review Board and Data Safety Monitoring Committee. All SAEs and AEs that require withdrawal of a patient from the study should be reported immediately and no later than 48 hours from the time study personnel learn of the event. All other events should be reported within 10 days of the study personnel learning of the event.

### 6.4.1 - Follow-up of AEs

AEs must be followed until the have resolved or have become stable with no resolution expected. Follow up of AEs must be recorded in medical record and on the case report forms.

## 6.5 - Expected AEs that do not require expedited reporting

There are numerous complications that could result form the surgical procedure itself. If any of these occurred, they will be assessed by the PI and provided the appropriate grade and attribution. Reporting will follow the guidelines detailed above.

## 6.6 - Investigator Reporting to the FDA

Serious adverse events (SAEs) that are unlisted/unexpected, and at least possibly associated to the drug, and that have not previously been reported in the Investigators brochure, or reference safety information document should be reported promptly to the Food and Drug Administration (FDA) by telephone or by fax. Fatal or life threatening SAEs that meet the criteria for reporting to the FDA must be reported to the FDA within 7 calendar days after awareness of the event. All other SAEs that meet the criteria for reporting to the FDA must be reported to the FDA within 15 calendar days after awareness of the event. A clear description of the suspected reaction should be provided along with an assessment as to whether the event is drug or disease related.

## 6.7 - Data Saftey Monitoring Plan

The data and safety monitoring plan will involve the continuous evaluation of safety, data quality and data timeliness. Investigators will conduct continuous review of data and patient safety at their regular Disease Group meetings (at least monthly) and the discussion will be documented in the minutes. The PI of the trial will review toxicities and responses of the trial where applicable at these disease center meetings and determine if the risk/benefit ratio of the trial changes. Frequency and severity of adverse events will be reviewed by the PI and compared to what is known about the agent/device from other sources; including published literature, scientific meetings and discussions with the sponsors, to determine if the trial should be terminated before completion. Serious adverse events and responses will also be reviewed by the OSUCCC Data and Safety Monitoring Committee (DSMC). The PI will also submit a quarterly progress that will be reviewed by the committee per the DSMC plan. All reportable Serious Adverse Events (SAE) will also be reported to the IRB of record as per the policies of the IRB.

|  | Within 4 weeks prior to catheter Placement | Day of surgery, catheter placement, and infusion | Day after surgery | Post-infusion day 1 | Day 7 | Day 14 | Day 28 | Week 8 | Week 12 | Week 16 | Week 22 | Week 30 |
| --- | --- | --- | --- | --- | --- | --- | --- | --- | --- | --- | --- | --- |
| Informed Consent | X |  |  |  |  |  |  |  |  |  |  |  |
| Inclusion/Exclusion  Criteria | X |  |  |  |  |  |  |  |  |  |  |  |
| Pathology | X | X |  |  |  |  |  |  |  |  |  |  |
| Medical History | X |  |  | X |  |  | X | X | X | X | X | X |
| Concomitant Meds | X | X |  | X |  |  | X | X | X | X | X | X |
| Physical Exam and Vital Signs | X | X | X | X |  | X | X | X | X | X | X | X |
| Neurological examination | X | X | X | X |  | X | X | X | X | X | X | X |
| MMSE | X | X | X | X |  | X | X | X | X | X | X | X |
| Performance Status Determination | X | X | X | X |  | X | X | X | X | X | X | X |
| CBC with differential and platelets, | X | X | X | X | X | X | X | X | X | X |  |  |
| Comprehensive Metabolic Profile | X | X | X | X | X | X | X | X | X | X |  |  |
| Pregnancy Testing | X |  |  |  |  |  |  |  |  |  |  |  |
| MRI Brain^1^ | X |  |  |  |  |  |  | X |  | X |  | X |
| AE monitoring | X | X | X | X | X | X | X | X | X | X | X | X |
| CT Brain |  | X |  |  |  |  |  |  |  |  |  |  |

7 – Study Calendar

1. MRI will be performed after catheter removal.

# 8 - Clinical Response and Endpoint Definitions

## 8.1 - Primary endpoint

The primary endpoint is to establish the dose-limiting toxicity and maximum tolerated dosage of convection enhanced delivery of carboplatin. DLT is defined as grade 3 or 4 toxicity as defined by the National Cancer Institute – Clinical Trials Group version 4.0 that is attributable to the study drug or protocol. In addition, grade 2 intracranial hemorrhage is a DLT. The maximum tolerated dosage is defined as the dose level below that causing DLT in at least two patients.

## 8.2 - Secondary endpoints

Secondary endpoints include evaluations of efficacy and drug distribution. Evaluations of efficacy will include assessing the six-month progression free survival and median progression free survival, as well as the radiographic response rate. Volume of distribution of infusate will be evaluated radiographically by post-infusion MRI. Six month progression free survival is defined as the proportion of patients with stable disease at 6 months from surgery. Progression free survival is defined as time between surgery and earliest sign of disease progression or death. Overall survival is defined as the time from surgery until death.

Radiographic and Clinical Response Criteria

8.2.1 - Neurological Examination Status (compared to baseline assessment)

| Better | Must be on stable or decreasing dosage of corticosteroids |
| --- | --- |
| Stable | Failure to qualify as better or worse |
| Worse | Includes patients requiring increasing steroid dosage to remain stable |

If a patient requires a higher dose of steroid to maintain a stable neurological status, the patient will be considered worse for the purpose of response determination.

Patients who are clinically worse but whose steroids have been decreased at the time of deterioration will be considered stable until reassessed again at the baseline steroid dosage.

### 8.2.2 - Radiographic Assessment compared to baseline MRI for patients with measurable disease

| CR | Total disappearance of all tumor with the patient off or only on adrenal replacement maintenance corticosteroids |
| --- | --- |
| PR | ≥50% reduction in product of perpendicular diameters of contrast enhancement or mass with no new lesions with the patient being on stable or decreased corticosteroid dosage |
| STAB | Failure to qualify for CR, PR, or PROG |
| PROG | >25% increase in product of perpendicular diameter of contrast enhancement or appearance of new lesions |

CR – Complete Response

PR – Partial Response

STAB – Stable disease

PROG – Progression of disease

### 8.2.3 - Evaluation of Best Overall Response

The best overall response is the best response recorded from the start of the treatment until disease progression/recurrence (taking as reference for progressive disease the smallest measurements recorded since the treatment started). The patient's best response assignment will depend on the achievement of both measurement and confirmation criteria.

| Neuro  Status | MRI and/or CT status | | | |
| --- | --- | --- | --- | --- |
|  | CR | PR | STAB | PROG |
| Better | CR | PR | STAB | Unknown* |
| Same |  |  |  | PROG |
| Worse | Unknown* | | |  |

If objective response is unknown, than will reimage in 4 weeks and classify according to:

| Neuro  Status | MRI and/or CT status | | | |
| --- | --- | --- | --- | --- |
|  | CR | PR | STAB | PROG |
| Better | CR | PR | STAB | Unknown* |
| Same |  |  |  | PROG |
| Worse | Progression | | |  |

### 8.2.4 - Confirmation of response

Objective tumor response (Complete Response - CR or Partial Response - PR) must be confirmed by a second assessment approximately 4 weeks from the first evidence of objective tumor response. Subsequent imaging for patients with response will be performed every 6 weeks until progression of disease is determined. In the case of stable disease, follow-up measurements must have met the stable disease criteria at least once after study entry at a minimum interval of 6 weeks (see section 12.2.2).

# 9 - Statistical Considerations

A standard phase I study design utilizing dose escalation as defined in section 2.0 will be used. A maximum of 42 patients will be accrued. Descriptive statistics of response/efficacy and tolerance of treatments will be used. Only patients who received study drug will be considered for endpoint analysis. Patients that did not receive study treatment (catheter placement and convection enhance delivery of carboplatin) will be replaced at the current dose cohort.

# 10 - References

1. Davis, F.G., et al., *Primary brain tumor incidence rates in four United States regions, 1985-1989: a pilot study.* Neuroepidemiology, 1996. **15**(2): p. 103-12.

2. Bidros, D.S. and M.A. Vogelbaum, *Novel drug delivery strategies in neuro-oncology.* Neurotherapeutics, 2009. **6**(3): p. 539-46.

3. Adamson, C., et al., *Glioblastoma multiforme: a review of where we have been and where we are going.* Expert Opin Investig Drugs, 2009. **18**(8): p. 1061-83.

4. Muldoon, L.L., et al., *Chemotherapy delivery issues in central nervous system malignancy: a reality check.* J Clin Oncol, 2007. **25**(16): p. 2295-305.

5. Teodorczyk, M. and A. Martin-Villalba, *Sensing invasion: cell surface receptors driving spreading of glioblastoma.* J Cell Physiol, 2010. **222**(1): p. 1-10.

6. Debinski, W. and S.B. Tatter, *Convection-enhanced delivery for the treatment of brain tumors.* Expert Rev Neurother, 2009. **9**(10): p. 1519-27.

7. El-Zein, R., Bondy, M., Wrensch, M., *Epidemiology of brain tumors.* 2005: p. 3-18.

8. Behin, A., et al., *Primary brain tumours in adults.* Lancet, 2003. **361**(9354): p. 323-31.

9. Stupp, R., et al., *Radiotherapy plus concomitant and adjuvant temozolomide for glioblastoma.* N Engl J Med, 2005. **352**(10): p. 987-96.

10. Stupp, R., et al., *Chemoradiotherapy in malignant glioma: standard of care and future directions.* J Clin Oncol, 2007. **25**(26): p. 4127-36.

11. Stupp, R., et al., *Effects of radiotherapy with concomitant and adjuvant temozolomide versus radiotherapy alone on survival in glioblastoma in a randomised phase III study: 5-year analysis of the EORTC-NCIC trial.* Lancet Oncol, 2009. **10**(5): p. 459-66.

12. Kelland, L., *The resurgence of platinum-based cancer chemotherapy.* Nat Rev Cancer, 2007. **7**(8): p. 573-84.

13. Chadwick, K.H., et al., *An analysis of the interaction of a platinum complex and radiation with CHO cells using the molecular theory of cell survival.* Int J Radiat Biol Relat Stud Phys Chem Med, 1976. **30**(6): p. 511-24.

14. Chater, S., et al., *Differential effects of ionizing radiation and platinum-derivative chemotherapy on apoptotic pathways in testicular germ cells.* Int J Radiat Biol, 2007. **83**(4): p. 269-78.

15. Stewart DJ, M.J., Eapen L, et al., *Cisplatin and radiation in the treatment of tumors of the central nervous system: pharmacological considerations and results of early studies.* Int J Radiat Biol, 1993. **28**: p. 531-542.

16. Yapp, D.T., et al., *The potentiation of the effect of radiation treatment by intratumoral delivery of cisplatin.* Int J Radiat Oncol Biol Phys, 1998. **42**(2): p. 413-20.

17. Yunhua B., X.Y., Jindong W., et al., *Cisplatin as a radiosensitizer in clinical practice: a pilot study.* Tumori, 1991. **77**: p. 21-24.

18. Kaneko, S., N.R. Clendenon, and M. Kartha, *Experimental study in a rat brain tumor by combined treatment with cis-DDP and irradiation.* Neurol Med Chir (Tokyo), 1983. **23**(12): p. 917-23.

19. Anonymous, *Cisplatin does not enhance the effect of radiation therapy in malignant gliomas. EORTC Brain Tumor Group.* Eur J Cancer, 1991. **27**(5): p. 568-71.

20. Olivi, A., et al., *Interstitial delivery of carboplatin via biodegradable polymers is effective against experimental glioma in the rat.* Cancer Chemother Pharmacol, 1996. **39**(1-2): p. 90-6.

21. Brem, H., *Polymers to treat brain tumours.* Biomaterials, 1990. **11**: p. 699-701.

22. Emerich, D.F., et al., *Injectable chemotherapeutic microspheres and glioma II: Enhanced survival following implantation into deep inoperable tumors.* Pharm Res, 2000. **17**(7): p. 776-81.

23. Sheleg, S.V., et al., *Local chemotherapy with cisplatin-depot for glioblastoma multiforme.* J Neurooncol, 2002. **60**(1): p. 53-9.

24. Emerich, D.F., S.R. Winn, and R.T. Bartus, *Injection of chemotherapeutic microspheres and glioma. IV: Eradicating tumors in rats.* Cell Transplant, 2002. **11**(1): p. 47-54.

25. Bobo, R.H., et al., *Convection-enhanced delivery of macromolecules in the brain.* Proc Natl Acad Sci U S A, 1994. **91**(6): p. 2076-80.

26. Bouvier, G., et al., *Direct delivery of medication into a brain tumor through multiple chronically implanted catheters.* Neurosurgery, 1987. **20**(2): p. 286-91.

27. Groothuis, D.R., *The blood-brain and blood-tumor barriers: a review of strategies for increasing drug delivery.* Neuro Oncol, 2000. **2**(1): p. 45-59.

28. Bidros, D.S., J.K. Liu, and M.A. Vogelbaum, *Future of convection-enhanced delivery in the treatment of brain tumors.* Future Oncol, 2009. **6**(1): p. 117-25.

29. Rousseau, J., et al., *Efficacy of intracerebral delivery of carboplatin in combination with photon irradiation for treatment of F98 glioma-bearing rats.* Int J Radiat Oncol Biol Phys, 2009. **73**(2): p. 530-6.

30. Rousseau, J., et al., *Enhanced survival and cure of F98 glioma-bearing rats following intracerebral delivery of carboplatin in combination with photon irradiation.* Clin Cancer Res, 2007. **13**(17): p. 5195-201.

31. Sampson, J.H., et al., *Intracerebral infusion of an EGFR-targeted toxin in recurrent malignant brain tumors.* Neuro Oncol, 2008. **10**(3): p. 320-9.

32. Aghi, M.K. and E.A. Chiocca, *Phase ib trial of oncolytic herpes virus G207 shows safety of multiple injections and documents viral replication.* Mol Ther, 2009. **17**(1): p. 8-9.

33. Yapp, D.T., et al., *Cisplatin delivery by biodegradable polymer implant is superior to systemic delivery by osmotic pump or i.p. injection in tumor-bearing mice.* Anticancer Drugs, 1998. **9**: p. 791-796.

34. Thomale, U.W., et al., *Local chemotherapy in the rat brainstem with multiple catheters: a feasibility study.* Childs Nerv Syst, 2009. **25**(1): p. 21-8.

35. Carson, B.S., Sr., et al., *New approach to tumor therapy for inoperable areas of the brain: chronic intraparenchymal drug delivery.* J Neurooncol, 2002. **60**(2): p. 151-8.

36. Guarnieri, M., et al., *Flexible versus rigid catheters for chronic administration of exogenous agents into central nervous system tissues.* J Neurosci Methods, 2005. **144**(2): p. 147-52.

37. Wu, Q., et al., *Section on tumors: Young Investigator Award: Local release of carboplatin via an Alzet mini-osmotic pump prolongs survival in a rat brainstem tumor model.* Clin Neurosurg, 2004. **51**: p. 332-9.

38. Biston, M.C., et al., *Cure of Fischer rats bearing radioresistant F98 glioma treated with cis-platinum and irradiated with monochromatic synchrotron X-rays.* Cancer Res, 2004. **64**(7): p. 2317-23.

39. Rousseau, J., et al., *Efficacy of intracerebral delivery of cisplatin in combination with photon irradiation for treatment of brain tumors.* J Neurooncol, 2010. **98**(3): p. 287-95.

40. Barth, R.F., et al., *Boron neutron capture therapy of brain tumors: enhanced survival and cure following blood-brain barrier disruption and intracarotid injection of sodium borocaptate and boronophenylalanine.* Int J Radiat Oncol Biol Phys, 2000. **47**(1): p. 209-18.

41. Yang, W., et al., *Convection enhanced delivery of carboplatin in combination with radiotherapy for the treatment of brain tumors.* J Neurooncol, 2010.

42. Guarnieri, M., et al., *Flexible versus rigid catheters for chronic administration of exogenous agents into central nervous system tissues.* Journal of Neuroscience Methods, 2005. **144**: p. 147-152.

43. Strege, R.J., et al., *Toxicity and cerebrospinal fluid levels of carboplatin chronically infused into the brainstem of a primate.* J Neurooncol, 2004. **67**(3): p. 327-34.

44. Degen, J.W., et al., *Safety and efficacy of convection-enhanced delivery of gemcitabine or carboplatin in a malignant glioma model in rats.* J Neurosurg, 2003. **99**(5): p. 893-8.

45. Tange, Y., et al., *Novel antitumor effect of carboplatin delivered by intracerebral microinfusion in a rat malignant glioma model.* Neurol Med Chir (Tokyo), 2009. **49**(12): p. 572-9.

46. Rousseau, J., et al., *Efficacy of intracerebral delivery of cisplatin in combination with photon irradiation for treatment of brain tumors.* J Neurooncol, 2009: p. DOI 10.1007/s11060-009-0074-3.

47. Carey, J.P., et al., *Ototoxicity of carboplatin delivered locally in a monkey brainstem.* Int J Toxicol, 2005. **24**(6): p. 443-9.

48. Kunwar, S., et al., *Phase III randomized trial of CED of IL13-PE38QQR vs Gliadel wafers for recurrent glioblastoma.* Neuro Oncol. **12**(8): p. 871-81.

49. Kunwar, S., et al., *Direct intracerebral delivery of cintredekin besudotox (IL13-PE38QQR) in recurrent malignant glioma: a report by the Cintredekin Besudotox Intraparenchymal Study Group.* J Clin Oncol, 2007. **25**(7): p. 837-44.

50. Wu, Q., et al., *Section on tumors: Young Investigator Award: Local release of carboplatin via an Alzet mini-osmotic pump prolongs survival in a rat brainstem tumor model.* Clinical Neurosurgery, 2004. **51**: p. 332-339.

# 11 - Appendices

## Appendix A- Mini-Mental State Examination (MMSE)
